# Advanced Separation Technologies for Cascade Biorefinery Valorization of *Capsicum* Seed Residues

**DOI:** 10.3390/foods15203699

**Published:** 2026-10-17

**Authors:** Gilbert Rodríguez, Abel José Rodríguez-Yparraguirre, Carlos Diego Rodríguez-Yparraguirre, Elza Aguirre, William E. López-Rodríguez, Janet E. Muñoz-Agreda, Soledad Quezada Berru, Cesar Moreno-Rojo, Guisela P. Carbajal-Romero, María de Fátima Arevalo-Oliva, Judy Muñoz-Saenz, Alyssa Hidalgo

**Affiliations:** 1 Instituto de Investigación Tecnológica Agroindustrial (IITA), Universidad Nacional del Santa, Av. Universitaria s/n, Nuevo Chimbote 02712, Ancash, Peru; eaguirre@uns.edu.pe (E.A.); 201012001@uns.edu.pe (W.E.L.-R.); jmunoz@uns.edu.pe (J.E.M.-A.); gcarbajal@uns.edu.pe (G.P.C.-R.); 2024818024@uns.edu.pe (M.d.F.A.-O.); 2 Department of Agroindustry and Agronomy, Faculty of Engineering, Universidad Nacional del Santa, Chimbote 02712, Ancash, Peru; cmoreno@uns.edu.pe; 3 Doctoral Program in Science and Engineering, Graduate School, Universidad Nacional de Trujillo, Trujillo 130101, La Libertad, Peru; cdrodriguezy@unitru.edu.pe; 4 Faculty of Engineering, Universidad César Vallejo, Chimbote 02712, Ancash, Peru; squezadab@ucvvirtual.edu.pe; 5 Servicio Nacional de Adiestramiento en Trabajo Industrial (SENATI), Independencia 15311, Lima, Peru; jmunoz@senati.edu.pe; 6 Dipartimento di Scienze per gli Alimenti, la Nutrizione e l’Ambiente (DeFENS), Università degli Studi di Milano, Via Celoria 2, 20133 Milan, Italy; alyssa.hidalgovidal@unimi.it

**Keywords:** green extraction, byproduct utilization, ultrasound-assisted extraction

## Abstract

*Capsicum* seed waste is an underutilized agro-industrial biomass with considerable potential for the recovery of oils, proteins, phenolic compounds, capsaicinoids, peptides, and other value-added fractions. This study evaluates advanced separation technologies for valorization in a cascade biorefinery, with an emphasis on extraction yield, selectivity, product functionality, sustainability, and technological scalability. A systematic literature search was conducted in Scopus and Web of Science, and the retrieved records were merged and processed using R (version 4.6.1) in RStudio (version 2026.01.2+418) Study selection and presentation were aligned with the PRISMA protocol, resulting in 82 studies included for thematic analysis. The evidence indicates that ultrasound-assisted, enzymatic, aqueous oleosome, pressure-based, membrane, and chromatographic approaches can improve the recovery and selective separation of valuable seed-derived fractions. The reported experimental results include an oleosome recovery of 66.96% by weight, with a potential recovery approaching 90% by weight, and a 73.5% increase in extraction recovery after enzyme-assisted processing. Protein modification and fractionation have also demonstrated opportunities for the development of functional ingredients, bioactive fractions, and biomaterials. However, limited pilot-scale validation, process integration, standardization, techno-economic feasibility, and life-cycle assessments constrain industrial implementation. Future research must prioritize scalable, closed-loop cascade strategies to minimize energy use, solvent consumption, and environmental footprint while maximizing biomass conversion.

## 1. Introduction

The expansion of agro-food production has intensified the generation of residual biomass throughout harvesting, processing, and post-processing operations, creating persistent challenges for resource management and environmental sustainability [1]. Although these streams are frequently treated as low-value waste, they retain organic matter and functional constituents that represent an opportunity for recovering materials and compounds with economic potential [2]. Their inadequate disposal can contribute to greenhouse-gas emissions, pollution, and unnecessary losses of water, energy, land, and other resources embedded in food production [3]. Consequently, current food bioprocessing strategies increasingly seek to shift from linear disposal toward circular models in which residual biomass is converted into value-added products through integrated recovery and biorefinery approaches [4].

The industrial processing of *Capsicum* fruits includes operations such as drying, grinding, slicing, sauce production, spice manufacture, and the recovery of pigments and pungent compounds [5]. These processes separate seeds and other anatomical fractions from the edible or targeted portions, generating heterogeneous residual streams whose composition depends on cultivar, maturity, processing conditions, and intended product [6]. Seeds are commonly treated as a secondary by-product despite retaining substantial amounts of lipids, proteins, dietary fiber, and bioactive constituents [7]. Studies on discarded seeds from pepper-processing operations have demonstrated their nutritional and functional potential, supporting their consideration as a recoverable biomass rather than a disposal burden [8,9]. This composition makes *Capsicum* seed residues suitable feedstocks for integrated extraction, separation, and cascade valorization strategies [10].

*Capsicum* seed residues constitute a chemically diverse biomass containing lipids, proteins, dietary fiber, and a range of minor compounds with functional properties [7]. Lipids generally represent one of the most valuable fractions, with linoleic acid predominating in the seed oil, while proteins and fiber provide opportunities for nutritional ingredients and subsequent processing of defatted residues [11]. The seeds also contain phenolic compounds, carotenoids, and capsaicinoids with antioxidant and other biological activities, although their concentrations vary according to species, cultivar, maturity, and processing conditions [12]. Furthermore, tocopherols and phytosterols contribute to the functional quality and oxidative stability of the lipid fraction [13]. This compositional diversity supports sequential recovery rather than single-product extraction, providing a basis for cascade biorefinery strategies [14].

Conventional valorization of *Capsicum* seed residues has predominantly focused on oil recovery, despite the presence of proteins, phenolics, pigments, antioxidants, and other valuable constituents within the same biomass [15]. Mechanical pressing may leave substantial quantities of extractable compounds in the residual cake, whereas solvent-based processes increase recovery but introduce concerns associated with solvent consumption, separation, and environmental burden [16]. Moreover, conventional extraction frequently provides limited selectivity, resulting in complex extracts that require additional purification [17]. Thermal exposure and prolonged processing may also affect oxidation-sensitive or thermolabile constituents, while the remaining solid fractions are commonly underexploited [18]. These limitations indicate that recovering a single target product is insufficient for maximizing the resource potential of *Capsicum* seed residues [19].

**Figure 1:**
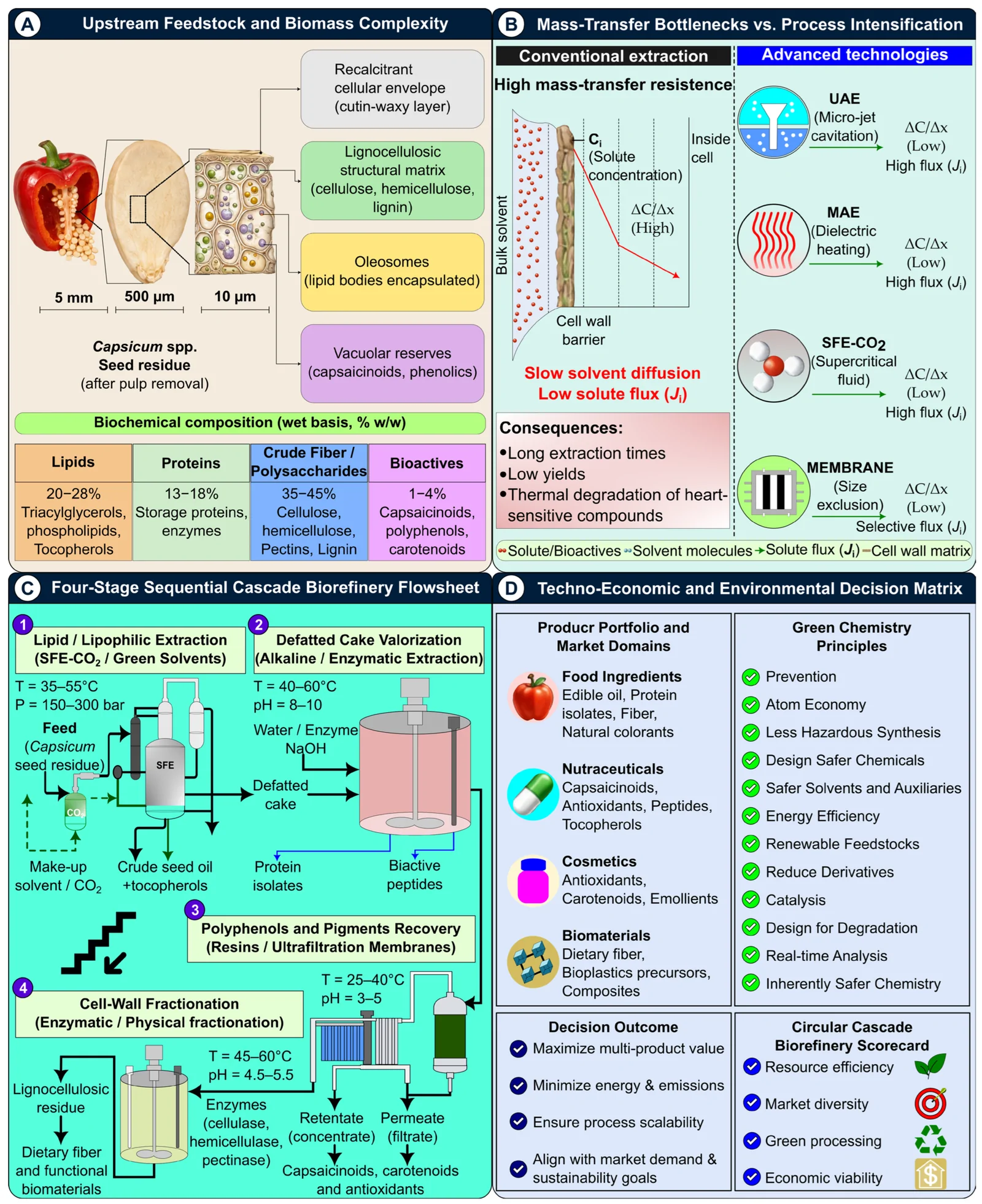
Integrated multi-scale conceptual framework governing the cascade biorefinery valorization of *Capsicum* seed residues. (**A**) Structural complexity, cell wall barriers, and biochemical composition of the seed matrix. (**B**) Mass-transfer mechanisms contrasting conventional extraction bottlenecks with advanced green technology intensification. (**C**) Four-stage sequential processing flowsheet for the fractionated recovery of lipophilic compounds, proteins, polyphenols, and dietary fiber. (**D**) Techno-economic, green chemistry, and circular economy decision matrix for market-driven applications.

Despite increasing interest in the recovery of bioactive compounds from *Capsicum* seeds, the available literature remains fragmented among oil extraction, recovery of individual compounds, and characterization of isolated fractions [8]. Existing studies have generally assessed extraction technologies according to the yield or quality of a specific product, providing limited insight into how successive separation operations could be combined to recover multiple fractions from the same biomass [20,21]. To address this gap, an integrated multi-scale conceptual framework for the cascade biorefinery valorization of Capsicum seed residues is proposed (Figure 1).

Despite the various processing approaches, there remains a scarcity of comparative studies addressing mass transfer efficiency, selectivity, energy and solvent requirements, product preservation, scalability, and subsequent valorization [22,23]. This fragmentation prevents a comprehensive assessment of *Capsicum* seed residues as multicomponent biorefinery feedstocks [22]. Therefore, the central question addressed in this review is: How can advanced separation technologies be integrated into cascade biorefinery pathways to maximize the recovery, quality, and valorization of multiple fractions from *Capsicum* seed residues?

This review aims to critically examine advanced separation technologies for the recovery and valorization of multiple components from *Capsicum* seed residues within a cascade biorefinery framework. Particular attention is given to the mechanisms underlying mass-transfer intensification, extraction and separation performance, selectivity, recovery efficiency, preservation of bioactive compounds, solvent and energy requirements, and technological scalability.

## 2. Materials and Methods

### 2.1. Review Design and Methodological Approach

This review was designed as a critical and technology-oriented assessment of advanced separation technologies for the valorization of *Capsicum* seed residues. A structured literature search was conducted to identify relevant studies addressing extraction, separation, fractionation, recovery, and subsequent utilization of valuable compounds from these residues. The PRISMA 2020 framework was used to improve transparency in the identification, screening, and selection of the literature considered for the review, rather than to establish a systematic review or meta-analytical study design [24]. The completed PRISMA checklist is available as (Supplementary Material Table S1). The selected evidence was subsequently organized according to the technological pathway, target fraction, separation principle, process performance, sustainability considerations, and potential integration into cascade biorefinery schemes. This approach enables a comparative and critical interpretation of the available knowledge while identifying technological gaps and opportunities for integrated *Capsicum* seed valorization.

### 2.2. Eligibility Criteria

Eligible publications investigated *Capsicum* seeds or seed-derived residues and reported information on extraction, separation, fractionation, purification, recovery, or valorization of their constituent compounds. Peer-reviewed primary research articles written in English were included when they provided sufficient methodological and experimental detail for technological comparison. Relevant fractions included oils, proteins, phenolics, carotenoids, capsaicinoids, tocopherols, phytosterols, dietary fiber, and other value-added components.

Evidence from other *Capsicum* matrices, including pericarp, pulp, whole fruits, or aerial tissues, was considered only when the reported technology showed clear potential for application to seed-derived residues. Such publications were treated as complementary technological evidence rather than direct validation of seed-residue performance. Reviews, conference proceedings, editorials, book chapters, records unrelated to seed valorization, and articles lacking sufficient technological or processing information were excluded.

### 2.3. Information Sources

The literature search was conducted using Scopus and Web of Science, selected for their broad coverage of peer-reviewed research in food science, bioprocessing, separation technologies, and biomass valorization. Both databases were searched for studies addressing *Capsicum* seed residues and technologies associated with the recovery and valorization of their constituent fractions. The search was restricted to publications from 2022 to 2026, in accordance with the predefined eligibility and selection criteria applied throughout the review process. This publication period was maintained consistently during database retrieval, screening, eligibility assessment, and final study selection.

### 2.4. Search Strategy

The search strategy was structured around three conceptual fields corresponding to the scope of the review: raw material, technology, and valorization. The first field included terms related to *Capsicum* species and seed-derived materials; the second encompassed extraction, recovery, separation, fractionation, purification, and emerging process-intensification technologies; and the third incorporated terminology associated with valorization, biorefinery, value-added products, bioactive compounds, functional ingredients, and circular bioeconomy. These fields were combined using the Boolean operator “AND”, whereas synonymous or closely related terms within each field were connected using “OR”. Searches were performed within the title, abstract, and keywords of indexed records.

The search equation in Scopus and Web of Science was established as follows: (“*Capsicum*” OR “*Capsicum annuum*” OR “*Capsicum frutescens*” OR “*Capsicum chinense*” OR “*Capsicum baccatum*” OR “*Capsicum pubescens*” OR pepper OR “chili pepper” OR “chilli pepper” OR “hot pepper” OR “pepper seed*” OR “chili seed*” OR “chilli seed*” OR “*Capsicum* seed*”) AND (extraction OR recovery OR separation OR fractionation OR purification OR isolation OR “ultra-sound-assisted extraction” OR “ultrasound assisted extraction” OR “microwave-assisted extraction” OR “microwave assisted extraction” OR “supercritical fluid extraction” OR “supercritical CO2” OR “enzyme-assisted extraction” OR “enzyme assisted extraction” OR “pulsed electric field*” OR “ohmic heating” OR “pressurized liquid extraction” OR “accelerated solvent extraction” OR “deep eutectic solvent*” OR “green extraction” OR “emerging extraction technolog*” OR “membrane separation” OR “membrane filtration” OR adsorption OR chromatography) AND (oil OR oils OR “seed oil*” OR lipid* OR fat* OR “fatty acid*” OR triglyceride* OR triacylglycerol* OR oleoresin* OR “oil extraction” OR “oil recovery” OR valorization OR valorisation OR biorefinery OR “cascade valorization” OR “cascade valorisation” OR “waste valorization” OR “waste valorisation” OR “by-product valorization” OR “by-product valorisation” OR “circular bioeconomy” OR “circular economy” OR “value-added product*” OR “value added product*” OR “high-value compound*” OR “bioactive compound*” OR “functional ingredient*” OR nutraceutical* OR “food ingredient*”).

### 2.5. Selection Process

**Figure 2:**
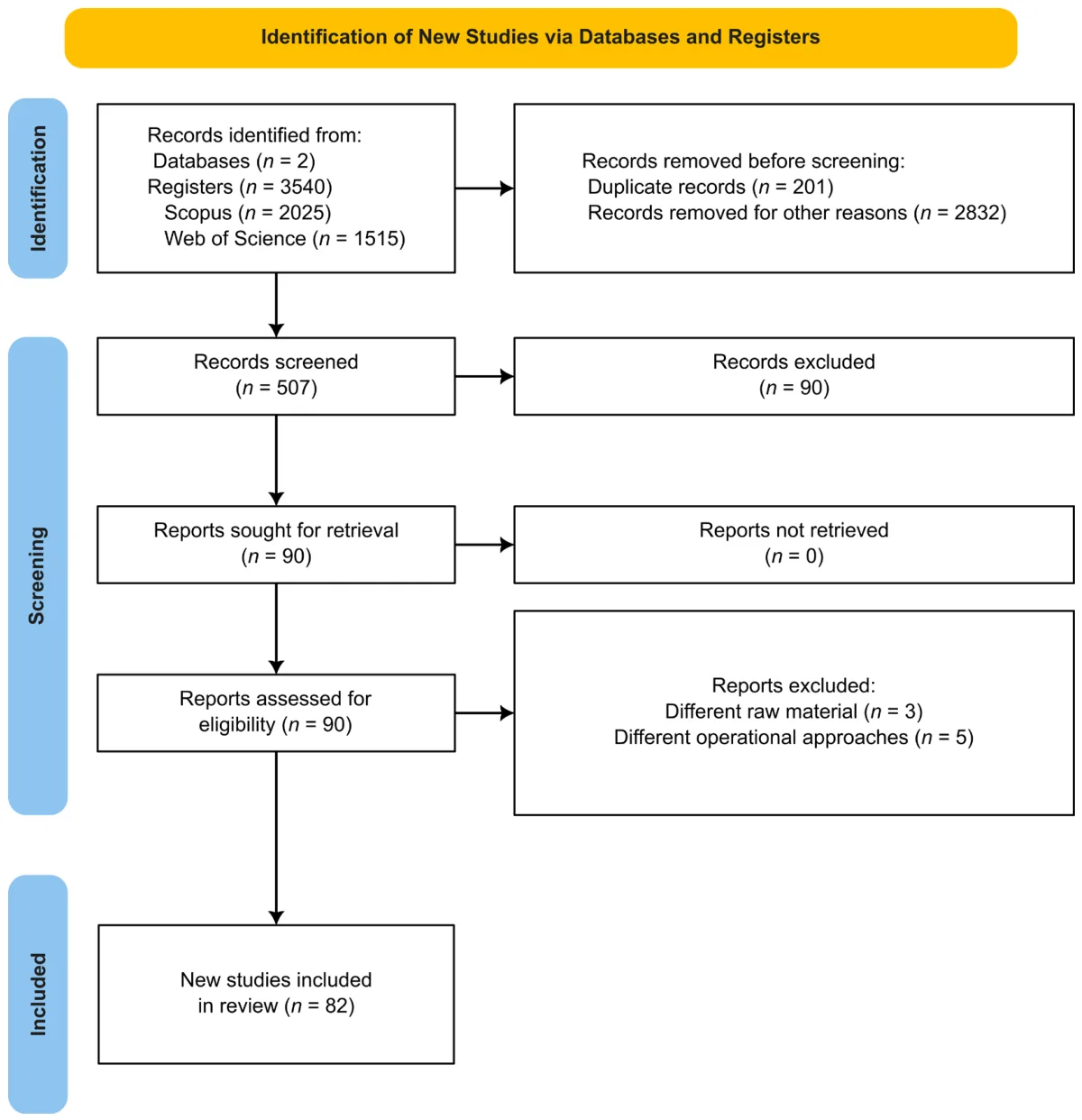
PRISMA flow diagram of the literature search, screening, eligibility criteria, and study selection process for *Capsicum* seed valorization.

The process of identifying and selecting studies was carried out through independent searches in Scopus and Web of Science, applying filters for publication year (2022–2026), document type (article), and open access. Initially, 2025 documents were identified in Scopus, of which 792 fell within the established period, 683 were articles, and 393 met the open access criteria. Initially, 1515 documents were retrieved from Web of Science, of which 636 were open access, 343 fell within the period 2022–2026, and 315 were articles. Integration of both databases using R (version 4.6.1, R Foundation for Statistical Computing, Vienna, Austria) in RStudio (version 2026.01.2+418, Posit Software, PBC, Boston, MA, USA) generated 708 records, of which 201 duplicates were removed, resulting in 507 unique records for the PRISMA identification phase (https://estech.shinyapps.io/prisma_flowdiagram/, accessed on 27 August 2026). During the screening by title and abstract, 90 studies were considered potentially eligible, and 417 were excluded. All 90 articles were retrieved for full-text evaluation. Subsequently, eight studies were excluded for not meeting the established experimental criteria, including three that did not use the raw material of interest and five that did not present operational results compatible with the objective of the review. Finally, 82 articles met all eligibility criteria and were included in the qualitative and thematic analysis (Figure 2).

### 2.6. Data Collection Process

Data were extracted from the selected studies using a structured framework designed to support comparison across the different technologies and valorization pathways. Information was collected on *Capsicum* species or cultivar, seed origin, pretreatment conditions, extraction or separation technology, operating parameters, solvent or processing medium, target compounds, recovery or extraction yield, purity, and relevant quality attributes. Where reported, energy requirements, solvent consumption, process duration, scalability, and environmental considerations were also recorded. Particular attention was given to the relationship between processing conditions and the recovery or preservation of valuable fractions, including lipids, proteins, phenolics, carotenoids, capsaicinoids, tocopherols, phytosterols, and fiber. The extracted information was subsequently organized according to technological category and valorization pathway to facilitate the critical comparison developed in Section 3, Section 4, Section 5, Section 6, Section 7, Section 8 and Section 9.

### 2.7. Data Items

The data items considered in the analysis were selected to characterize both the technological performance and the valorization potential of *Capsicum* seed residues. The extracted variables included raw material characteristics, pretreatment conditions, extraction or separation method, processing medium, temperature, time, solid-to-liquid ratio, and other relevant operating parameters. Performance indicators comprised extraction or recovery yield, selectivity, purity, composition, and retention of bioactive constituents. Additional information concerning energy demand, solvent requirements, solvent recovery, process complexity, scalability, and environmental performance was recorded when available. The potential applications of the recovered fractions were also documented, particularly for oils, proteins, phenolics, carotenoids, capsaicinoids, tocopherols, phytosterols, fiber, and other value-added products. These data items provided the basis for comparing individual technologies and evaluating their integration into cascade biorefinery pathways.

### 2.8. Synthesis Methods

The evidence was synthesized through a qualitative and comparative approach, given the substantial heterogeneity among the selected studies in terms of *Capsicum* species, seed characteristics, processing conditions, extraction methods, analytical procedures, and reported performance indicators. The literature was organized into thematic groups covering feedstock characteristics, mass-transfer barriers, primary extraction, advanced separation and fractionation, cascade biorefinery pathways, and sustainability and scale-up considerations. Within each group, technologies were compared according to recovery efficiency, selectivity, product quality, bioactive preservation, solvent and energy requirements, and technological maturity when such information was available. Rather than pooling numerical results statistically, the synthesis emphasized relationships between processing conditions and outcomes, technological advantages and limitations, and opportunities for process integration. This approach enabled the identification of recurring patterns, methodological inconsistencies, and research gaps relevant to the development of integrated *Capsicum* seed biorefineries.

## 3. Capsicum Seed Residues as Multicomponent Biorefinery Feedstocks

### 3.1. Origin and Characteristics of Capsicum Seed Residues

*Capsicum* seed residues should be regarded as multicomponent biorefinery feedstocks rather than as single-purpose sources of vegetable oil [8,25]. Their heterogeneous matrix contains lipid, protein, fiber, and minor bioactive fractions whose simultaneous presence creates opportunities for sequential recovery and product diversification [26]. The relative abundance of these constituents varies considerably with species, cultivar, maturity, geographical origin, processing conditions, and storage history, influencing both their technological accessibility and potential applications [27,28]. From a biorefinery perspective, this compositional complexity is advantageous because different fractions can be targeted through complementary separation mechanisms instead of being recovered through a single extraction step [29]. Consequently, mapping the composition and distribution of valuable constituents is essential for designing integrated processing schemes that maximize resource recovery, minimize residual biomass, and yield multiple value-added products [8].

The studies summarized in Table 1 differed substantially in *Capsicum* species and cultivars, plant material or fraction, pretreatment conditions, extraction media, analytical methods, and reporting units. Consequently, the quantitative values presented for seeds and associated fruit fractions should be interpreted within the experimental context of each study rather than as directly comparable measurements. The table is therefore intended to provide an overview of compositional diversity, major bioactive constituents, and their potential relevance for biorefinery valorization. Numerical data are included to illustrate the range of reported compositions and bioactive responses under the specific conditions used in each investigation, without implying equivalent analytical or processing conditions across studies.

### 3.2. Proximate Composition

The proximate composition indicates that *Capsicum* seeds are characterized by a substantial concentration of extractable and structurally valuable components, although their distribution varies markedly among species and cultivars [21]. In a large-scale characterization of 233 accessions representing nine *Capsicum* species, seed oil content ranged from 10.8 to 35.9%, with linoleic acid accounting for an average of 76.0% of the principal fatty acids, demonstrating that lipid recovery represents a consistent valorization route despite compositional variability [54]. This potential is reinforced by discarded *Capsicum annuum* seeds from pepper processing, which contained 18.30% protein, 11.04% oil and 60.96% dietary fiber, indicating that oil extraction can be integrated with subsequent recovery of protein- and fiber-rich fractions rather than treating the defatted residue as waste [55].

### 3.3. Lipids and Fatty Acids

Lipids represent one of the most valuable and consistent fractions in *Capsicum* seeds, although their abundance varies substantially within the genus. Studies report seed oil contents of 10.8–35.9% in nine *Capsicum* species, with linoleic acid predominating, reaching 81% in *Capsicum chinense*, indicating a strong polyunsaturated character [54]. In *Capsicum annuum*, cold-pressed oils from Podravka and Slavonka seeds were found to contain 75.4–77.7% linoleic acid, while supercritical CO_2_ extraction yielded 74.0–76.0%, demonstrating that the extraction technology caused only minor changes in fatty acid distribution [56]. Similarly, roasting red pepper seeds did not substantially alter their fatty acid profile, reinforcing their suitability for lipid recovery [46].

Despite the relatively high oil content reported across *Capsicum* species, the variability in lipid yield indicates that seed genotype and processing conditions may be more influential than extraction technology alone. The comparatively similar linoleic acid proportions obtained by cold pressing and supercritical CO_2_ extraction suggest that the latter does not necessarily provide a major advantage in preserving fatty acid composition, although it may offer greater control over extraction selectivity and operate without the use of conventional organic solvents [54,56]. From a biorefinery perspective, this distinction is important because extraction performance should not be evaluated exclusively according to oil yield or fatty acid composition, but also according to the possibility of recovering additional compounds from the defatted matrix. Cold pressing may therefore be attractive for relatively simple and scalable oil production, whereas supercritical CO_2_ may become more advantageous when selective recovery and product quality are prioritized. The limited changes observed after roasting further indicate that thermal pretreatment does not necessarily compromise the nutritional lipid profile; however, its effects on oxidation and the subsequent recovery of minor bioactive compounds should be considered before industrial implementation [46].

### 3.4. Proteins and Amino Acids

Protein represents a particularly relevant fraction of *Capsicum* seeds, as its value depends not only on concentration but also on amino acid composition and functionality. In bell pepper seeds, protein isolates contained 39.35% essential amino acids, with leucine (9.68 g/100 g), lysine (5.26 g/100 g), and threonine (4.79 g/100 g) among the predominant essential amino acids, while glutamic acid reached 14.11 g/100 g, indicating a nutritionally and functionally relevant profile [57]. On the other hand, it was demonstrated that ultrasound treatment modified the functional properties of seed protein isolates and that their hydrolysates exhibited antioxidant activity in vitro, extending their potential beyond nutritional applications to functional ingredients, and showing a yield of 6.05 g/100 g, a protein content of 83.93 g/100 g, and an oil absorption of 5.63 g/g [58]. Therefore, protein recovery after lipid extraction could generate a second, high-value stream instead of a residual fraction.

The protein fraction should therefore be considered as a complementary product stream rather than as a competing target to lipid recovery. The high proportion of essential amino acids reported for bell pepper seed protein indicates that the nutritional value of the recovered fraction can be retained even when the seeds are primarily considered an oil source [57]. However, protein functionality appears to be more sensitive to processing than fatty acid composition. Ultrasound treatment can modify protein structure and improve functional properties, while subsequent hydrolysis may further increase antioxidant activity, suggesting that the value of the protein fraction depends on the degree of processing and the intended application. This creates an important trade-off: intensive treatments may improve solubility, functionality, or bioactivity, but they may also increase processing requirements and affect protein structure [58].

### 3.5. Phenolic Compounds and Antioxidant Constituents

Phenolic compounds represent a valuable bioactive fraction of *Capsicum* seed residues because their antioxidant potential depends not only on concentration but also on molecular composition and interactions with other phytochemicals. In *Capsicum annuum* seeds, hydro-methanolic extraction identified 45 compounds, including phenolic acids, flavonoids, and capsaicinoids, while phenolics reached 193.7 mg GAE/g DW and were associated with measurable radical-scavenging activity [59]. Enzyme-assisted extraction provides an additional strategy for modifying this profile: cellulase treatment of jalapeño seeds released phenolic compounds while generating aggregates of approximately 660.9 nm, illustrating that enzymatic disruption can alter both recovery and functionality [60]. Similarly, yellow bell-pepper residues yielded extracts containing up to 19.55 mg TE/g DW by FRAP, confirming that residual fractions retain antioxidant functionality after primary processing [15]. Therefore, compositional characterization and bioactivity assessment should be integrated when defining the functional potential of *Capsicum* seed-derived fractions [61]. Such sequential recovery can increase resource utilization while reducing the amount of residual biomass requiring disposal, thereby connecting chemical composition with the broader objective of integrated *Capsicum* residue valorization [15].

The differences among these approaches indicate that phenolic recovery cannot be interpreted solely from the total phenolic content reported by each study. Solvent-based extraction can provide broad chemical recovery, whereas enzyme-assisted extraction may increase the accessibility of compounds that remain associated with disrupted cell-wall structures [59,60]. The formation of aggregates after cellulase treatment further suggests that enzymatic processing may modify the physical organization of the recovered material, which could influence its subsequent functional performance and downstream separation [60]. Importantly, the antioxidant capacity measured in residual pepper fractions demonstrates that valuable compounds remain after primary processing, but comparisons based on FRAP, radical-scavenging assays, or total phenolic content should be interpreted cautiously because these measurements reflect different aspects of antioxidant behavior [15,59].

### 3.6. Carotenoids and Capsaicinoids

Capsaicinoids are among the most characteristic bioactive constituents of *Capsicum*, and their reliable determination is essential for evaluating the quality and potential applications of pepper-derived fractions [62]. Analytical control therefore becomes essential when seed residues undergo extraction, concentration, or formulation, where changes in matrix composition can affect measurement accuracy. Recent electrochemical approaches demonstrate the feasibility of rapid monitoring: an AuNPs@Fe_3_O_4_-based sensor quantified capsaicinoids in oil over 0.10–10.00 ng/mL, achieving a detection limit of 0.02 ng/mL and $R^{2}=\;0.9978$ [63]. Similarly, an MXene–titania–Nafion sensor detected capsaicin down to 25 nM and was successfully applied to real pepper samples [64]. Analytical sensitivity consequently becomes more than a characterization requirement: it contributes directly to reproducibility, standardization, and quality assurance throughout the valorization chain [63].

The analytical approaches reported in these studies illustrate a broader distinction between conventional compositional characterization and process-oriented monitoring. The very low detection limits achieved by electrochemical sensors indicate that these technologies can provide rapid and sensitive measurements, which may be particularly useful for monitoring capsaicinoid concentrations during extraction, concentration, and formulation [63,64]. Nevertheless, analytical sensitivity alone does not determine suitability for industrial implementation. Sensor response can be affected by matrix composition, electrode fouling, selectivity, and calibration requirements, whereas chromatographic methods generally provide more comprehensive compound identification and quantification.

### 3.7. Tocopherols, Phytosterols, and Other Minor Compounds

Minor compounds contribute to the nutritional, oxidative, and sensory properties of *Capsicum* seed-derived fractions. Recent experimental analysis of cold-pressed sweet pepper seed oil reported 53.5–57.4 mg/100 g of γ-tocopherol across two harvest seasons, together with approximately 1.0–1.1 g/100 g of unsaponifiable compounds, confirming the relevance of minor lipid constituents in seed oil [65]. The same oil contained 19 volatile compounds, with (E)-β-ocimene representing approximately 40% of the detected volatile fraction, demonstrating the contribution of minor compounds to aroma characteristics [65]. These approaches can strengthen compositional fingerprinting and quality control of seed-derived fractions. Integrating predictive models with chromatographic measurements can therefore strengthen chemical fingerprinting and reduce uncertainty during the characterization of complex *Capsicum* fractions intended for food or ingredient applications [66]. Consequently, advanced chromatographic fingerprinting can support quality control within a biorefinery, particularly when seed-derived oils or aromatic fractions are considered as potential value-added products [67].

The presence of tocopherols, unsaponifiable compounds, and volatile constituents highlights an additional challenge in *Capsicum* seed valorization: minor compounds may contribute disproportionately to product quality despite representing a small fraction of the total biomass. The reported γ-tocopherol content and unsaponifiable fraction indicate that cold-pressed oils can retain nutritionally relevant minor constituents, while the volatile profile suggests that processing conditions may also determine the sensory and commercial attributes of the recovered oil [65]. This means that refining or purification should not be optimized exclusively for the removal of undesirable components, since excessive processing could also reduce desirable minor constituents. In this context, chromatographic fingerprinting provides greater compositional resolution than single-marker analysis and may be particularly useful for detecting batch-to-batch variation and changes caused by processing [65,66,67].

### 3.8. Variability Associated with Species, Cultivar, Maturity, Processing, and Storage

The composition of *Capsicum* seed residues varies with cultivar, maturity, extraction history, and storage, affecting the reproducibility of downstream separation. Experimental evaluation of red pepper seed oils obtained by cold pressing, ethanol extraction, and ultrasound-assisted extraction showed significant differences in oxidative stability during accelerated storage at 60 °C for 6 weeks, demonstrating that extraction method influences subsequent deterioration [13,68]. In another study, γ-irradiation of red pepper seeds at 2.5–10 kGy reduced microbial loads while progressively increasing free fatty acids from 0.98 to 1.43% and peroxide values from 0.78 to 1.30 meq O_2_/kg oil at the highest dose [13]. Drying should be considered an active processing variable capable of influencing subsequent recovery, rather than merely a preliminary operation [69]. Establishing chemical fingerprints according to species or cultivar can reduce variability between production batches and facilitate the selection of processing conditions appropriate for specific seed-derived streams [70]. Thus, cultivar selection and drying conditions should be considered jointly when defining feedstock specifications, allowing the biorefinery to maintain more consistent chemical inputs and improve the reproducibility of downstream recovery processes [71].

## 4. Structural Barriers and Mass-Transfer Limitations

### 4.1. Cellular Architecture of Capsicum Seeds

The cellular architecture of *Capsicum* seeds can restrict mass transfer because the seed coat consists of an outer epidermal layer with species-dependent anticlinal and periclinal cell-wall patterns, including reticulate, cerebelloid, and reticulate–cerebelloid sculptures [72]. Experimental evidence further demonstrates the functional importance of coat integrity: seeds with an intact coat showed 82% final germination, whereas seeds with the coat removed reached 37%; similarly, radical emergence decreased from 55.56% to 23.00% [73]. From a downstream perspective, pressure-assisted defatting of pepper seed meal yielded 20.5% protein isolates, compared with 22.8% using conventional solvent defatting, while increasing oil-holding capacity by 40% and emulsifying ability by 160% [74]. That is why, through experimental analyses, it is demonstrated that structural integrity is directed towards accessibility and recovery.

### 4.2. Cell-Wall Composition and Permeability

The cell wall of *Capsicum* seeds is characterized by a cellulose-rich polysaccharide matrix that limits solvent penetration and restricts the release of intracellular compounds. In Habanero pepper seeds (*Capsicum chinense*), cellulose complexes accounted for more than 43% of the seed composition, while capsaicinoids, flavonoids, and tocopherols were reported within the cell-wall matrix [75]. Consequently, enzymatic hydrolysis can increase wall permeability by disrupting these structural polymers. Cellulase-assisted extraction increased recovery by 73.5% at 60 °C for 150 min, whereas only a 1.42% increase was obtained at 45 °C for 30 min [75]. These results agree with the fiber-rich characteristics of pepper seed by-products, confirming that cell-wall disruption is essential for improving compound accessibility [76].

### 4.3. Intracellular Localization of Target Compounds

The intracellular localization of target compounds in *Capsicum* seeds further limits their accessibility during extraction. Histochemical observations have identified numerous protein bodies within endosperm vacuoles, confirming that proteins are stored in discrete intracellular structures rather than freely distributed throughout the matrix [77]. In Habanero seeds (*Capsicum chinense*), capsaicinoids, flavonoids, and tocopherols are reported as compounds embedded within the cell-wall-associated matrix, requiring enzymatic disruption for improved release [75]. Accordingly, cellulase-assisted extraction achieved a maximum total phenolic content of 337.96 mg GAE/L at 30 °C, 2500 UI/L, and 150 min [2]. In *Capsicum annuum* seeds, roasting at increasing temperatures decreased capsaicin and dihydrocapsaicin contents, demonstrating their sensitivity to structural and thermal disruption [46].

### 4.4. External and Internal Mass-Transfer Resistances

Mass transfer during *Capsicum* seed extraction involves external resistance at the solid–liquid interface and internal diffusion through the seed matrix. Microwave-assisted extraction of capsaicin from *Capsicum annuum* seeds achieved a yield of approximately 1.89% (18.88 mg/g) at 600 W using a 30 mL/g solvent-to-solid ratio, demonstrating the effect of intensified energy input on compound release [78]. In contrast, ultrasound-assisted extraction of red pepper seed oil with ethanol produced an oil yield of 16.80%, indicating that acoustic cavitation can improve solvent penetration and interfacial contact [79]. Furthermore, conventional and intensified extraction approaches applied to pepper seed residues have shown that solvent accessibility depends strongly on particle disruption and process conditions. Thus, reducing external resistance while shortening internal diffusion pathways is fundamental for improving recovery efficiency.

### 4.5. Effects of Particle Size, Moisture, and Pretreatment

Matrix accessibility in *Capsicum* extractions depends on balancing particle size, moisture, and pretreatments. Grinding green pepper to 80 mesh optimizes oleoresin yield (12.51%), whereas fine milling to 100 mesh causes agglomeration (11.90%) [80]; similarly, sweet paprika requires particles < 0.2 mm and 7.5% moisture for peak supercritical carotenoid recovery (45 MPa, 50 °C) [81]. Pre-drying below 8% moisture with grinding to 250–420 µm provides stable matrix handling [82], while enzymatic digestion (2500 UI/L cellulase, 60 °C, 150 min) increases yield by 73.5% through cell wall breakdown [75].

### 4.6. Relationship Between Matrix Disruption and Extraction Efficiency

Matrix disruption is closely related to extraction efficiency because weakening cellular structures facilitates the release of intracellular compounds. In *Capsicum chinense* seeds, cellulase-assisted extraction increases extraction yield by 73.5% at 60 °C for 150 min, while the increase was only 1.42% at 45 °C for 30 min, demonstrating the contribution of enzymatic disruption to recovery [75]. Ultrasound treatment of red pepper seed protein isolates for 5–20 min promotes protein unfolding and aggregate dissociation, with maximum improvements of 25% in solubility and 71.1% in oil-holding capacity [83]. Similarly, ultrasound-assisted extraction increases capsaicinoid extraction from *Capsicum baccatum* by 26% compared to conventional methods [9]. These findings demonstrate that structural disruption can improve compound accessibility and extraction yield; furthermore, they indicate that controlled matrix disruption can improve compound accessibility and extraction efficiency, although the degree of improvement depends on processing conditions and the target fraction.

## 5. Advanced Technologies for Primary Extraction and Recovery

### 5.1. Conventional Solvent Extraction as a Reference Process

Conventional solvent extraction remains an important benchmark because solvent composition strongly determines the selectivity and recovery of bioactive compounds from *Capsicum* seed matrices. Experimental comparison of green solvent systems for *Capsicum annuum* seeds showed that ethanol (50:50, *v*/*v*) provided higher phenolic recovery than individual solvents, with 88.60 mg/100 g dry weight obtained from green-pepper seeds compared with 22.30 mg/100 g from red-pepper seeds [84]. Direct extraction of *Capsicum chinense* seeds using water and cellulase further demonstrated that operating conditions strongly influenced compound recovery; the highest phenolic concentration reached 337.96 mg GAE/L at 30 °C, 2500 UI/L, and 150 min, whereas capsaicin reached 310.23 μg/mL at 45 °C, 250 UI/L, and 150 min [75].

Solvent composition and physicochemical properties significantly influence extraction selectivity by governing the preferential solubilization of target compounds; parameters such as solvent polarity, hydrogen-bonding capacity, and solvent–solute affinity determine the extent to which specific molecules migrate from the *Capsicum* matrix into the liquid phase. In this regard, polar solvent systems generally favor the recovery of phenolic compounds, whereas less polar media are better suited for lipophilic fractions, such as carotenoids and oils. Matrix characteristics also dictate solvent accessibility and mass transfer. Consequently, when comparing conventional and intensified extraction technologies in terms of selectivity, efficiency, and the preservation of bioactive compounds, the choice of solvent must be tailored to the chemical nature of the target fraction [49].

These findings demonstrate that conventional solvent extraction remains a useful benchmark—not necessarily because it is the most efficient or sustainable option, but because the solvent composition provides a controllable basis for understanding extraction selectivity. The markedly different phenolic compound concentrations obtained from green and red pepper seeds indicate that matrix composition can significantly influence solvent performance, complicating direct comparisons between extraction technologies when raw material characteristics are not standardized [84]. Likewise, the water–cellulase system illustrates that temperature, enzyme concentration, and extraction time can alter the preferential recovery of phenolic compounds and *capsaicinoids*, suggesting that selectivity is determined by interactions among solvent properties, matrix structure, and the polarity of the compounds of interest [75].

### 5.2. Mechanical Pressing

Mechanical pressing provides a solvent-free route for oil recovery from *Capsicum* seeds, although its efficiency depends on the lipid and structural characteristics of the raw material. Cold mechanical pressing of *Capsicum annuum*, *Capsicum baccatum*, *Capsicum chinense*, and *Capsicum pubescens* yields oil of 4.0–5.4 g/100 g, corresponding to a lipid recovery of 43.1–45.1%, with resulting oils containing between 67.9% and 75.6% polyunsaturated fatty acids [85]. Similarly, in the aqueous oleosome process, screw pressing of hydrated *Capsicum* seeds achieves an oleosome yield of 66.96% by weight, which could be increased to 90% by weight through process optimization [16].

Compared with solvent-based extraction, mechanical pressing offers an important advantage in terms of process simplicity and solvent avoidance, but the reported lipid recovery indicates that a considerable proportion of the seed oil may remain in the press cake [86]. This limitation becomes particularly relevant when the objective is to maximize total resource recovery rather than simply produce a crude oil fraction. The higher oleosome recovery achieved after optimization of the aqueous process suggests that seed hydration and disruption of the oil–protein matrix can substantially improve the accessibility of lipid structures [16]. 

### 5.3. Ultrasound-Assisted Extraction

Enhances mass transfer and matrix disruption through acoustic cavitation. In red bell pepper seed proteins, 5 to 20 min of sonication altered structural conformation and reduced particle size, increasing emulsifying activity by up to 49.4% and foaming properties by 71.1% [83]. Similarly, applying UAE to Guntur Sannam chili (100–300 W, 10–20 min, 50–100% methanol) showed that capsaicinoid recovery depends strongly on power and solvent concentration, confirming that ultrasonic treatments induce distinct structural modifications [86]. Optimization at 235 W, 17 min, and 68% methanol produced 107.32 mg/kg capsaicin and 56.93 mg/kg dihydrocapsaicin, while FT-IR and SEM confirmed structural disruption of the treated matrix [87]. However, excessive treatment can alter the matrix or compromise sensitive components; therefore, optimization must balance acoustic energy, treatment duration, and matrix response to maximize recovery without causing undesirable structural or chemical changes [88].

The evidence indicates that UAE provides a more versatile intensification mechanism than conventional extraction because acoustic cavitation can simultaneously improve solvent penetration, disrupt cellular structures, and modify the physical properties of recovered biomolecules [83,86,87]. However, the response is strongly dependent on treatment intensity and matrix composition. The improvement in protein emulsifying and foaming properties after relatively short sonication periods contrasts with the need to optimize power, solvent concentration, and exposure time for capsaicinoid recovery, demonstrating that there is no universal ultrasound condition for *Capsicum* residues [83,86]. Excessive acoustic energy may further promote structural modification beyond that required for compound release, which can compromise the quality or functionality of sensitive fractions [88].

### 5.4. Microwave-Assisted Extraction

Microwave-assisted extraction (MAE) intensifies mass transfer through rapid volumetric heating, enabling shorter treatment times and controlled energy input. Recent experimental optimization of MAE for *Capsicum annuum* demonstrated that microwave power, irradiation time, and solvent-to-solid ratio strongly influenced capsaicinoid recovery. Using ethyl acetate, optimized conditions of 220 W for 60 s and 30 mL/g produced 22.1 mg/g dry matter of capsaicin and 10.6 mg/g of dihydrocapsaicin, exceeding the corresponding acetone extraction [89]. Similarly, microwave extraction of *Capsicum annuum* var. *glabriusculum* optimized a combination of 77.84 min irradiation, 360 mL water, and 0.536 mm particle size, yielding an extract containing 504.26 mg GAE/100 g phenolics and 3.48 mg/g vitamin C [90]. Therefore, microwave-assisted operations should be integrated with compositional monitoring to identify conditions that simultaneously support efficient processing, bioactive preservation, and adequate stability of the resulting seed-derived material [91].

The results demonstrate that MAE can substantially shorten extraction times compared with conventional solid–liquid approaches, but its performance is highly dependent on the dielectric properties of the solvent, moisture content, particle size, and applied microwave energy. The markedly different optimum conditions reported for capsaicinoids and phenolic compounds indicate that microwave irradiation does not produce a single extraction regime applicable to all *Capsicum* targets [89,90]. Rapid volumetric heating can enhance solvent penetration and accelerate cell disruption, but excessive irradiation may increase local temperatures and potentially compromise thermolabile compounds or promote unwanted transformations.

### 5.5. Supercritical Fluid Extraction

Supercritical fluid extraction (SFE) using CO_2_ enables selective recovery of lipophilic compounds while reducing dependence on conventional organic solvents. In industrial *Capsicum annuum* waste, increasing temperature and adding ethanol as a co-solvent enhanced oleoresin recovery, while kinetic parameters were evaluated for process design [92]. Pilot-scale extraction of dry paprika demonstrated that pressure, temperature, and extraction time significantly influenced performance, with optimal conditions of 45 MPa, 50 °C, and 74 min producing a 10.05% extract yield and 4.21% total carotenoids [93]. Similarly, SC-CO_2_ extraction with ethanol increased fatty-acid recovery, whereas the highest carotenoid content was obtained at 45 MPa and 50 °C without co-solvent [94].

The evidence indicates that SFE provides a degree of selectivity that distinguishes it from conventional solvent extraction, particularly for lipophilic compounds such as carotenoids and fatty acids [92,93,94]. However, the reported results also demonstrate that selectivity is highly sensitive to pressure, temperature, extraction time, and the use of ethanol as a co-solvent. The observation that ethanol increased fatty-acid recovery whereas the highest carotenoid content was obtained without co-solvent illustrates an inherent trade-off between extraction yield and fraction selectivity [94]. This behavior can be explained by changes in solvent polarity and solute–matrix interactions under supercritical conditions, which modify the affinity of the extraction phase for compounds with different chemical properties.

### 5.6. Enzyme-Assisted Extraction

Enzyme-assisted extraction (EAE) enhances compound recovery by hydrolyzing structural polysaccharides of *Capsicum* seed cell walls, thereby improving matrix accessibility. In Habanero seeds (*Capsicum chinense*), cellulase treatment increased extraction yield by 73.5% at 60 °C for 150 min using 2500 IU/L enzyme, while total phenolic concentration increased by 79–87% at 30 °C after 120–150 min [75]. Under optimized conditions, capsaicin reached 310.23 μg/mL at 45 °C, 250 IU/L, and 150 min, whereas dihydrocapsaicin reached 167.72 μg/mL at 60 °C, 2500 IU/L, and 120 min [75]. In *Capsicum annuum*, cellulase treatment at 45 °C for 30–180 min further demonstrated its capacity to modify phenolic compounds and soluble carbohydrates, supporting EAE for selective seed fractionation [60].

The results obtained with EAE highlight the importance of adjusting enzymatic activity to the structural characteristics of the seed matrix and the physicochemical properties of the compounds of interest. Cellulase can enhance accessibility by hydrolyzing structural polysaccharides and weakening cell wall barriers, thereby facilitating the release of compounds that would otherwise be difficult for the extraction medium to access [60,75]. However, optimal conditions differed markedly among phenolic compounds, capsaicin, and dihydrocapsaicin, indicating that matrix disruption and compound release are not governed by a single mechanism; increasing enzyme concentration or temperature may improve the release of some compounds while offering limited additional benefits for others.

### 5.7. Pressurized Liquid and Accelerated Solvent Extraction

Pressurized liquid extraction (PLE), also termed accelerated solvent extraction (ASE), intensifies solid–liquid extraction by combining elevated temperature and pressure to improve solvent penetration and solute transfer. In red pepper waste, sequential processing using supercritical extraction followed by PLE enabled recovery of phenolic compounds, with ethanol concentration significantly affecting extraction yield, phenolic composition, and antioxidant activity [1]. For paprika (*Capsicum*), ASE optimized at 78 °C, 28 min, and two cycles produced an oleoresin with an ASTA color value of 847 and 57,200 ± 362 SHU, exceeding maceration and ultrasound extraction [2]. Another ASE study obtained 198.23 mg/g extract using water at 100 °C for 19.5 min, demonstrating the potential for rapid, solvent-efficient phenolic recovery [3].

PLE/ASE occupies an intermediate position between conventional solvent extraction and highly intensified technologies because it retains a solvent-based mechanism while using elevated temperature and pressure to accelerate mass transfer. The reported results demonstrate that this combination can reduce extraction time and improve recovery, but they also show that process performance is strongly dependent on solvent composition and operating temperature [1,2,3]. The high ASTA color and capsaicinoid values obtained by ASE indicate that the technology can be particularly effective when concentrated oleoresin is the desired product [2]. However, the use of water at elevated temperature producing substantial phenolic recovery suggests that organic solvents are not always necessary to achieve effective extraction [3].

### 5.8. Pulsed Electric Field and Ohmic-Assisted Extraction

Pulsed electric field (PEF) treatment can enhance mass transfer through electroporation while limiting extensive thermal exposure. In red bell pepper tissue, PEF applied at an electric field strength of 1.07 kV/cm and specific energy inputs of 1 and 3 kJ/kg reduced subsequent convective drying time by 31.6% and 35.1%, respectively [95]. Increasing the energy input also promoted structural modification, as electrical conductivity increased from 165 to 533 µS/cm. Under these conditions, carotenoid availability increased by approximately 30% at 1 kJ/kg and 48% at 3 kJ/kg, while antioxidant activity improved by about 15.2–30.8% compared with untreated material [95].

### 5.9. Deep Eutectic Solvents and Emerging Green Extraction Approaches

Deep eutectic solvents (DESs) and natural deep eutectic solvents (NADESs) provide adjustable, low-volatility means for recovering bioactive compounds from *Capsicum* matrices. Ultrasound-assisted extraction using choline chloride-urea achieves 18.88 mg/g of capsaicin at 600 W and a solvent-to-solid ratio of 30 mL/g, demonstrating the advantage of combining DES with process intensification [78]. In pepper crop residues, a choline chloride/1,6-hexanediol NADES containing 30 wt% water yielded 10.31 ± 0.16 mg of quercetin equivalents/g of dry biomass and outperformed hydroethanolic extraction for glycosylated flavonoids [96]. More recently, enzyme-assisted NADES extraction achieved 9.91 ± 0.13 mg/g of capsaicin under optimized conditions, demonstrating synergistic matrix depolymerization and improved mass transfer [97].

The evidence indicates that no single extraction technology consistently provides the best performance across all *Capsicum* target compounds. Conventional solvent extraction remains an important reference because solvent composition can be adjusted according to the polarity and chemical characteristics of the target compounds [75,84]. Mechanical pressing offers a solvent-free route for lipid recovery, although the reported lipid recovery of 43.1–45.1% indicates that a substantial fraction of the lipids may remain in the residual matrix [85]. UAE and MAE enhance extraction through different mechanisms and can achieve high recovery under optimized conditions, but their performance depends strongly on treatment intensity, solvent composition, and processing time [83,86,87,88,89,90,91]. SFE provides greater control over the recovery of lipophilic fractions through changes in pressure, temperature, and co-solvent addition, although the optimal conditions differ according to whether carotenoids or fatty acids are targeted [92,93,94]. EAE can improve matrix accessibility through enzymatic hydrolysis, while PLE/ASE combines elevated temperature and pressure to accelerate solvent-based extraction [1,2,3,60,75]. DESs and NADESs further expand the range of solvent systems available for selective recovery, although their performance remains dependent on solvent composition and water content [78,96,97].

### 5.10. Comparative Evaluation of Primary Extraction Technologies

Evaluating extraction technologies requires balancing operational metrics rather than optimizing single-compound yields. Because high gross yields often co-extract undesirable compounds, assessing *Capsicum* seed valorization must weigh recovery, selectivity, solvent use, and downstream compatibility. In cascade biorefineries, primary extraction efficiency should ultimately be judged by its overall footprint and impact on final fraction quality [15], with key technological parameters outlined in Table 2.

Drying conditions directly limit the efficiency of subsequent extraction stages. Moisture content, structural alterations, and bioactive stability dictate how accessible compounds remain within the plant matrix. Drying trials for *Capsicum chinense* reveal that different technologies modify physicochemical, functional, and bioactive properties, meaning no single method fits all scenarios. In a biorefinery workflow, pretreatment selection must align with the target downstream product. Fast moisture reduction is counterproductive if it degrades thermolabile compounds or alters functional properties needed later. Drying is an integral part of the primary recovery chain, not an isolated step. Its parameters must balance drying speed, energy costs, structural integrity, and the recovery potential of the subsequent extraction steps [26].

To ensure a consistent and transparent evaluation in Table 2, scalability was classified into three levels: low, medium, and high. Low scalability was assigned to extraction processes evaluated solely under laboratory conditions, without documented evidence of scale-up or operation under processing-relevant conditions. Medium scalability was assigned when the study demonstrated technical feasibility for larger-scale application, including the use of scalable equipment, compatible extraction media, or operating conditions that allowed for further process development. High scalability was assigned to technologies whose feasibility had been demonstrated beyond the laboratory scale, particularly through pilot, demonstration, field, or industrial applications. These predefined criteria were applied uniformly to all analyzed technologies to reduce subjectivity and improve comparability among the described extraction methods.

## 6. Advanced Separation, Fractionation, and Purification

### 6.1. Clarification and Solid–Liquid Separation

Clarification and solid–liquid separation are essential downstream operations for removing suspended particles and concentrating target fractions after *Capsicum* extraction. In an aqueous oleosome process, hydrated *Capsicum* seeds were mechanically pressed and the extract was centrifuged to separate cream, pellet, and serum fractions, achieving an oleosome extraction yield of 66.96 wt% [16]. Similarly, alkaline extraction of pepper seed proteins employed centrifugation at 10,000 rpm for 20 min to recover the protein-containing supernatant before subsequent fractionation [112]. In an integrated pepper-sauce waste process, gravity settling followed by centrifugation at 4000 rpm for 10 min recovered residual ethanolic extract, while subsequent centrifugation at 8000 rpm for 15 min removed insoluble impurities from the crude oil fraction [113].

The reported processes indicate that clarification is not merely a preparatory operation but a critical determinant of downstream recovery efficiency. Centrifugation provides rapid separation of suspended solids and phase-specific fractions, but its effectiveness depends on particle size, density differences, viscosity, and the characteristics of the extracted matrix [16,112,113]. The different centrifugation conditions reported across studies therefore reflect distinct separation objectives rather than interchangeable operating parameters. For example, lower centrifugal forces may be sufficient for recovering an extract phase, whereas higher forces are required to remove fine insoluble particles from crude oil [113].

### 6.2. Membrane-Based Separation

Membrane-based separation provides a selective downstream strategy for fractionating *Capsicum* seed extracts according to molecular size, while simultaneously reducing chemical requirements. In Yidu red pepper seeds, a two-stage ultrafiltration process was integrated using 10 and 5 kDa membranes with alkaline extraction and isoelectric precipitation, generating fractions above 10 kDa, between 5 and 10 kDa, and below 5 kDa [114]. Compared to conventional extraction, the ultrafiltration-assisted process increased protein recovery by 5.28% and reduced hydrochloric acid consumption by 19.48%. The above-10 kDa fraction also exhibited a 9.88% increase in solubility, a 78.66% increase in water-holding capacity, and a 22.69% increase in emulsification stability [114].

Membrane fractionation provides an important advantage over precipitation-based separation because molecular-size selectivity can be introduced without requiring repeated chemical modification of the extract. The sequential use of 10 and 5 kDa membranes demonstrates that ultrafiltration can generate compositionally differentiated protein fractions while simultaneously reducing acid consumption [114]. The improvement in solubility, water-holding capacity, and emulsification stability further indicates that membrane processing may influence not only fraction purity but also functional performance. However, membrane separation is inherently affected by concentration polarization and fouling, particularly when processing complex extracts containing proteins, polysaccharides, lipids, and phenolics.

### 6.3. Centrifugation and Phase Separation

Centrifugation and phase separation are critical downstream operations for separating oil-rich, aqueous, and insoluble fractions generated during *Capsicum* biomass processing. In an integrated valorization process, pepper sauce waste was subjected to centrifugation at 4000 rpm for 10 min to recover residual ethanolic extract, while subsequent centrifugation at 8000 rpm for 15 min removed insoluble impurities from the crude red pepper oil [113]. For aqueous extraction of *Capsicum* seeds, oleosomes were recovered after steeping and mechanical pressing, with separation losses identified as the main contributor to the process exergy loss; the experimental process achieved an oleosome extraction yield of 66.96 wt%, with optimization scenarios indicating a potential increase to 90 wt% [16].

Available evidence suggests that phase separation can constitute a significant bottleneck in *Capsicum* biorefinery processes, particularly when multiple valuable fractions are generated simultaneously. The fact that separation losses are the primary contributor to exergy loss during oleosome recovery demonstrates that the efficiency of downstream operations can significantly influence the overall sustainability of the process [16]. In this regard, centrifugal force can improve phase recovery, but the associated energy demand must be balanced against the additional product recovered—a distinction that highlights the difference between maximizing separation efficiency and maximizing process efficiency. Furthermore, centrifugation is particularly effective when there is a sufficient density difference between the phases, whereas membrane or adsorption technologies may be preferable when the compounds of interest remain dissolved in a single phase.

### 6.4. Adsorption and Resin-Based Recovery

Adsorption onto solid matrices provides a selective downstream route to concentrate capsaicinoids from complex *Capsicum* extracts. In *Capsicum annuum*, screening 19 heterogeneous adsorbents identified magnesium–aluminum azelate and bentonite as the most effective, yielding capsaicinoid recoveries of 73.5–91.6% and 68.4–70.8%, respectively [115]. Likewise, coupling alkaline eutectic solvent extraction with macroporous resin adsorption achieved 19.15 mg/g capsaicin, with SP850 outperforming XAD2 and XAD16N in both adsorption capacity and recovery efficiency [116].

Adsorption provides a higher degree of chemical selectivity than bulk separation techniques because the interaction between the target compounds and the adsorbent can be exploited to concentrate specific capsaicinoids from complex extracts [115,116]. The different performance of magnesium–aluminum azelate, bentonite, SP850, XAD2, and XAD16N demonstrates that adsorbent chemistry is a major determinant of recovery and that no single resin can be assumed to be optimal for all *Capsicum* matrices.

### 6.5. Chromatographic Separation and Purification

Chromatographic methods offer the resolution required to distinguish closely related capsaicinoids and verify fraction composition. Validating HPLC-DAD protocols across *Capsicum* accessions ensures accurate quantification, which is critical for process optimization; relying solely on total capsaicinoid content can mask shifts in individual compound profiles. Reliable determination further separates raw material variability from processing noise, establishing clear product specifications across biorefinery streams [117]. Characterizing pepper oleoresins requires sensitive methods to profile pungency and quality parameters. Optimized HPLC protocols improve spiciness evaluation, providing the analytical precision needed for seed-residue valorization. Because fraction value depends on consistent chemical profiling rather than yield alone, reliable chromatographic resolution enables tracking target capsaicinoids across sequential processing steps to verify end-product specifications [118].

Chromatography differs fundamentally from the other downstream technologies because its primary value is analytical resolution and high compositional control rather than bulk recovery. HPLC-DAD can distinguish individual capsaicinoids that may be masked when only total capsaicinoid concentration is considered, thereby providing a more reliable basis for quality specifications and process monitoring [117,118]. However, the high resolution of chromatographic methods is generally accompanied by greater solvent consumption, equipment requirements, and operating costs than bulk separation technologies.

### 6.6. Oleosome and Oil-Body Recovery

Oleosome and oil-body recovery enables the preservation of seed lipids within naturally structured oil–protein assemblies, offering an alternative to conventional free-oil extraction. Experimental characterization of sweet pepper seeds showed a lipid content of 19.57%, confirming their potential as an oil-rich residue suitable for downstream lipid recovery [119]. Cold pressing of seeds from four *Capsicum* species subsequently produced oil yields of 4.0–5.4 g/100 g, corresponding to 43.1–45.1% lipid recovery, while polyunsaturated fatty acids represented 67.9–75.6% of the recovered oils [85]. These findings indicate that oil-body-oriented processing can preserve nutritionally valuable lipid fractions and provide a feedstock for subsequent fractionation of functional lipids, proteins, and minor bioactive compounds.

Evidence suggests that oleosome-based recovery offers a fundamentally different strategy from conventional oil extraction, as it seeks to preserve lipids within natural oil–protein structures rather than disrupting the matrix to obtain free oil. The relatively moderate lipid recovery achieved via cold pressing indicates that conventional pressing may leave a significant fraction of lipids in the residual biomass, whereas oleosome recovery may allow for a more integrated separation of oil-associated structures [16,85]. However, higher recovery achieved under optimized conditions does not necessarily imply greater product value, given that the composition, stability, and downstream functionality of oleosome-rich fractions must also be considered. In this regard, preserving the native oil–protein interface may be advantageous for applications requiring structured lipids or emulsifying functionality, whereas free oil extraction may be preferable when the desired product consists of purified lipids.

### 6.7. Protein and Peptide Fractionation

Protein and peptide fractionation provides a pathway to convert *Capsicum* seed proteins into compositionally differentiated and potentially functional fractions. In pepper seeds, reverse micelle extraction yielded a protein fraction containing 78.75% albumin, while alkaline solubilization followed by acid precipitation generated a glutelin-dominated fraction, accounting for 65.33% of the recovered protein [112]. More recently, enzymatic digestion of *Capsicum annuum* var. *conoides* seed protein yielded a hydrolysate with a 26.4% yield, and LC-MS/MS analysis identified three novel peptides with immunomodulatory activity [120].

The reported results demonstrate that protein fractionation can transform a heterogeneous seed protein pool into fractions with substantially different compositions and potential functions [112,120]. Reverse micelle extraction favored albumin-rich material, whereas alkaline solubilization followed by acid precipitation produced a glutelin-dominated fraction, indicating that the separation mechanism strongly determines the chemical identity of the recovered proteins. Subsequent enzymatic hydrolysis introduces a second level of selectivity because peptide composition and bioactivity depend on both the original protein substrate and the specificity of the hydrolytic treatment [120]. This sequential approach therefore offers greater functional differentiation than recovering total protein alone. However, the identification of bioactive peptides does not automatically establish their suitability as food ingredients, since stability, digestibility, sensory effects, bioavailability, and safety require further validation.

### 6.8. Recovery and Purification of Phenolics, Carotenoids, and Capsaicinoids

The recovery and purification of phenols, carotenoids, and capsaicinoids require selective downstream conditions because these compounds differ markedly in polarity and stability. In red habanero pepper waste, supercritical fluid extraction with a cosolvent provided the highest overall recovery, yielding 14.9 mg/g of total capsaicinoids and 292.09 μg/g of total carotenoids [29]. Furthermore, experimental analysis showed that the combination of methanol/ethyl acetate/petroleum ether or aqueous ethanol followed by re-extraction with hexane/dichloromethane achieved carotenoid extraction efficiencies of 99.6% and 100%, respectively, while optimized saponification yielded over 97% carotenoid recovery [121]. In paprika, SC-CO_2_ at 50 °C and 45 MPa produced the highest carotenoid content, with capsanthin as the predominant pigment [94].

The contrasting recovery profiles reported for phenolics, carotenoids, and capsaicinoids demonstrate why a single downstream operation is unlikely to maximize the value of all *Capsicum* bioactive fractions simultaneously. The high recovery of carotenoids obtained after saponification and solvent re-extraction reflects the effectiveness of aggressive fraction-specific processing, but such conditions may be inappropriate when the objective is to preserve other sensitive compounds or obtain food-compatible extracts [121]. Similarly, the ability of SC-CO_2_ to concentrate carotenoids while recovering capsaicinoids illustrates the potential of controlling solvent polarity and operating conditions to direct fraction composition [29,94].

### 6.9. Selectivity and Purity of Recovered Fractions

The selectivity and purity of the recovered fractions determine their suitability for downstream applications in food, nutraceuticals, or analysis. From *Capsicum annuum* chili extracts, dispersive liquid–liquid microextraction (DLLME) selectively isolated capsaicin, dihydrocapsaicin, and nordihydrocapsaicin, providing relative recoveries of 92.0–108.0% and amplified fractions with >98.0% purity, suitable for high-purity analytical isolation [122]. For paprika biomass, pilot-scale supercritical CO_2_ extraction yielded an extract with an 87% carotenoid recovery and 56% unsaturated fatty acid recovery, supporting its use as a carotenoid- and lipid-rich fraction [93]. Conversely, an optimized carotenoid extraction-saponification protocol for *Capsicum annuum* achieved a carotenoid recovery >97% and identified 48 carotenoids, providing a highly selective analytical fraction for comprehensive pigment characterization [121].

Scalability of the separation and fractionation strategies summarized in Table 3 was classified as Low, Medium, or High using predefined criteria related to process scale, equipment requirements, and evidence of operation beyond laboratory conditions. Low scalability was assigned to procedures restricted to laboratory-scale separation or purification, particularly those involving analytical or multistep operations with limited scale-up evidence. Medium scalability was assigned to technologies showing potential for larger-scale implementation through adaptable equipment, food-compatible processing media, or application-oriented validation, although pilot-scale demonstration remained limited. High scalability was reserved for processes supported by pilot-scale operation, industrially established equipment, or direct evidence of successful scale-up.

## 7. Cascade Biorefinery Valorization of Capsicum Seed Residues

### 7.1. Principles of Cascade Biorefinery

The cascade biorefinery concept shifts *Capsicum* residue management from single-product extraction toward sequential resource utilization, in which the recovery of one fraction is designed to preserve or enhance the value of the remaining biomass. This approach was demonstrated experimentally in pepper-processing waste, where integrated separation generated hot pepper oil, a no-heat residue, no-heat oil, capsaicinoids, and capsaicin, with reported yields of 8.0%, 25.3%, 4.0%, 0.8%, and 0.4%, respectively [113]. For seed-derived streams, the same logic is particularly relevant because oil recovery does not exhaust the nutritional potential of the residue: protein and fiber were subsequently isolated from cold-pressed hot pepper seed oil by-product and incorporated into a low-fat salad dressing, demonstrating a transition from primary fraction recovery to food-ingredient development [123]. This sequential perspective is further supported by the experimental use of pepper seed protein in nanoemulsions, showing that recovered fractions can undergo additional functional upgrading rather than being treated as terminal by-products [57].

### 7.2. First-Stage Recovery: Oils and Lipid-Rich Fractions

Oil recovery represents a logical first stage in *Capsicum* seed biorefineries because lipid-rich fractions can be separated while leaving a protein- and fiber-containing meal available for subsequent valorization. Recent experimental evidence confirms that mechanical pressing provides a relatively mild route for this initial fractionation: seeds from four *Capsicum* species yielded 4.0–5.4 g oil/100 g, corresponding to 43.1–45.1% lipid recovery, while the recovered oils contained 67.9–75.6% polyunsaturated fatty acids [86]. Alternative intensification can accelerate recovery; ohmic heating produced 14.53 ± 0.4% oil at 3.5 V/cm for 4 min, compared with 16.57 ± 0.03% for conventional extraction, while increasing phenolic concentration to 28.95 mg GAE/g oil [126]. Importantly, the resulting press cake remains a valuable secondary feedstock, as demonstrated by protein and fiber isolation from hot pepper seed oil by-product [123].

### 7.3. Second-Stage Recovery: Proteins and Peptides

Protein recovery is a key second-stage operation because the matrix of defatted *Capsicum* seeds can be transformed from a waste meal into a functional protein resource, while subsequent hydrolysis expands its potential to yield bioactive peptides. Experimental fractionation of pepper seed proteins showed that albumin exhibited a 23.49% increase in emulsifying activity after high hydrostatic pressure (HHP) treatment, compared to 13.46% for glutelin, indicating that protein functionality can be selectively enhanced after recovery [112]. Similarly, HHP treatment of pepper seed protein isolate increased water-holding capacity, oil-holding capacity, foaming characteristics, and emulsifying activity index by up to 121.98%, 157.29%, 100.00%, and 265.78%, respectively [127]. Furthermore, enzymatic hydrolysis of chili seed protein under HHP improved antibacterial activity against *S. aureus* by 18.90%, demonstrating the transition of recovered protein towards peptide-based functional applications [128].

### 7.4. Third-Stage Recovery: Phenolics and Antioxidant Compounds

The third stage of *Capsicum* seed biorefinery can target phenolics and other antioxidant compounds that remain distributed within the defatted or previously fractionated matrix. Their recovery is particularly relevant because antioxidant functionality provides an additional food-ingredient value beyond the primary oil and protein streams. In an experimental study using *Capsicum annuum* seeds discarded by a harissa-processing facility, hydro-methanolic extraction identified 45 phytochemicals and produced an extract containing 193.7 mg GAE/g DW of phenolics and 25.1 mg QE/g DW of flavonoids, with DPPH, reducing, and chelating activities of 45.0, 61.3, and 79.0 µg/mL, respectively [59]. Complementary analysis of Australian bell-pepper seeds also showed greater phenolic concentrations in seed fractions than in corresponding pulp, supporting their recovery as antioxidant food ingredients [41]. Thus, this stage converts residual phytochemicals into a distinct functional stream rather than treating them as losses.

### 7.5. Recovery of Carotenoids, Capsaicinoids, Tocopherols, and Phytosterols

Valorizing Capsicum seed lipids depends on preserving minor bioactive constituents that enhance oil functionality. Thermal treatments applied to *Capsicum annuum* alter capsaicinoid levels and volatile profiles, indicating that oil composition reflects both raw material origin and processing history. In cascade biorefineries, thermal management during extraction dictates whether these high-value compounds are retained or degraded. Extraction parameters must therefore balance overall lipid yield against the preservation of capsaicinoids and other bioactive molecules that differentiate seed-derived oils [129].

### 7.6. Valorization of Fiber- and Polysaccharide-Rich Fractions

Fiber- and polysaccharide-rich streams represent an important downstream opportunity because their value can be increased through conversion into functional food ingredients rather than disposal or low-value uses. Recent experimental characterization of *Capsicum* seeds reported fiber contents of 49.0–55.2 g/100 g dry matter across four species, confirming the substantial structural fraction remaining after lipid recovery [85]. For polysaccharide-oriented valorization, industrial *Capsicum* residue yielded 8.09% pectin under acidic extraction, with 65% degree of methylation and 75% galacturonic acid, demonstrating the potential of residual biomass as a source of food-grade hydrocolloids [10]. At the product level, pepper seed press-cake was incorporated into dipping sauces at 5–10 g/100 g, improving rheological and sensory properties, illustrating that fiber-rich residual solids can be directly upgraded into formulated foods rather than undergoing complete chemical fractionation [130].

### 7.7. Valorization of Residual Biomass

Residual biomass represents the final stream of the cascade and should be redirected toward biological or energetic conversion rather than discarded. Experimental digestion of pepper-processing waste demonstrated a biogas potential of 687 L/kg dry matter and a biomethane potential of 401 L/kg dry matter, while the polyphenolic fraction progressively decreased during digestion, indicating that residual bioactive compounds can be transformed during energy recovery [131]. A complementary study using pepper waste after polyphenolic extraction achieved 464 ± 25 NL CH_4_/kg VS in batch digestion, whereas semi-continuous operation with thermal pretreatment produced 160 NL/kg VS, demonstrating the feasibility of converting post-extraction solids into bioenergy [132]. More recently, industrial *Capsicum* residue showed a maximum volatile fatty acid production of 462.09 mg/L, supporting acidogenic conversion as another downstream route [10].

Diverting residual *Capsicum* biomass into sequential recovery streams enhances cascade biorefinery efficiency. Cellulase-assisted extraction of habanero seeds yielded 337.96 mg GAE/L phenolics, 310.23 µg/mL capsaicin, and 167.72 µg/mL dihydrocapsaicin, significantly reducing nitric oxide production to 22.56% [75]. Similarly, ultrasound-assisted extraction (8 min) of industrial red habanero waste recovered 26% oleoresin (7 mg/g capsaicinoids, 4 mg GAE/g phenolics) with 95% less energy than reflux methods [107]. Further valorization of chili seed proteins into nanoemulsions (319 ± 2 nm) and antioxidant peptides demonstrates the potential of residual solids to deliver functional food ingredients beyond lipids and capsaicinoids [57,133].

### 7.8. Integrated Sequential Processing Configurations

Integrated sequential processing requires that the order of unit operations preserve the functionality of the recovered fractions while creating suitable intermediates for subsequent steps. In pepper processing waste, an integrated experimental setup combined two ethanol extraction cycles with subsequent C18 flash chromatography, using 95% ethanol for 3 h per extraction and a 130 g column with a sample concentration of 100 mg/mL, demonstrating how downstream purification can be incorporated without discarding intermediate streams [113]. For seed-derived fractions, sequential processing can extend beyond separation to food formulation: protein and fiber recovered from chili pepper seed oil byproduct were incorporated into low-fat dressing systems, with consistency indexes ranging from 6.82 to $22.32\;Pa\cdot s^{n}$ and oxidative induction periods from 5.11 to 7.04 h [123]. These indicators suggest that process integration should be evaluated based on the compatibility and functionality of successive flows, rather than the performance of isolated operations.

The effectiveness of a cascade biorefinery depends on whether each upstream operation preserves the suitability of the remaining material for subsequent recovery steps. Mild oil extraction can retain protein- and fiber-rich fractions for further processing, as demonstrated by their isolation and incorporation into food formulations [123]. In contrast, excessive thermal exposure may alter capsaicinoids and other thermosensitive compounds, reducing their downstream value [129]. Selective extraction based on compound polarity can improve fraction differentiation and facilitate subsequent recovery, as reported for supercritical fluid extraction followed by ethanol-based pressurized extraction [22]. Ultrasound-assisted processing may also reduce energy demand and extraction time while maintaining valuable bioactive fractions [107]. Therefore, the main trade-off lies between maximizing immediate recovery and preserving the chemical and functional integrity required for later valorization stages.

Likewise, integrated sequential processing enables the multi-product valorization of Capsicum waste by tailoring extraction stages to compound polarity. Combining supercritical fluid extraction (60 °C, 25 MPa) with pressurized liquid extraction using ethanol successfully separated carotenoid-rich oleoresin from phenolics [22]. Similarly, supercritical extraction of jalapeño waste confirmed that higher temperatures and ethanol as a co-solvent enhanced oleoresin yield while preserving its bioactivity [113]. An integrated process for chili sauce waste yielded 8.0% (*v*/*w*) of hot red chili oil, 25.3% (*w*/*w*) of residue, 4.0% (*v*/*w*) of non-pungent oil, 0.8% (*w*/*w*) of capsaicinoids, and 0.4% (*w*/*w*) of capsaicin [57]. Finally, proteins from chili seeds enabled the production of nanoemulsions with droplet sizes of 319 ± 2 nm, supporting the subsequent valorization of defatted fractions and enhancing overall resource-use efficiency within cascade biorefineries.

A conceptual mass-flow framework was established using 100 kg of dry *Capsicum* seed residues as a reference basis. Based on reported seed composition, this biomass contains approximately 9.2–12.0 kg of lipids, 19.8–23.5 kg of protein, and 49.0–55.2 kg of fiber [85]. Cold pressing can recover about 4.0–5.4 kg of oil, leaving approximately 94.6–96.0 kg of press cake for subsequent fractionation [85]. This residual stream can then undergo protein recovery [123], followed by extraction of phenolic and other bioactive compounds [59], while the remaining fiber-rich fraction can be directed toward functional ingredients or further conversion. The final residual biomass can subsequently be valorized through biological or energetic routes [131,132].

**Figure 3:**
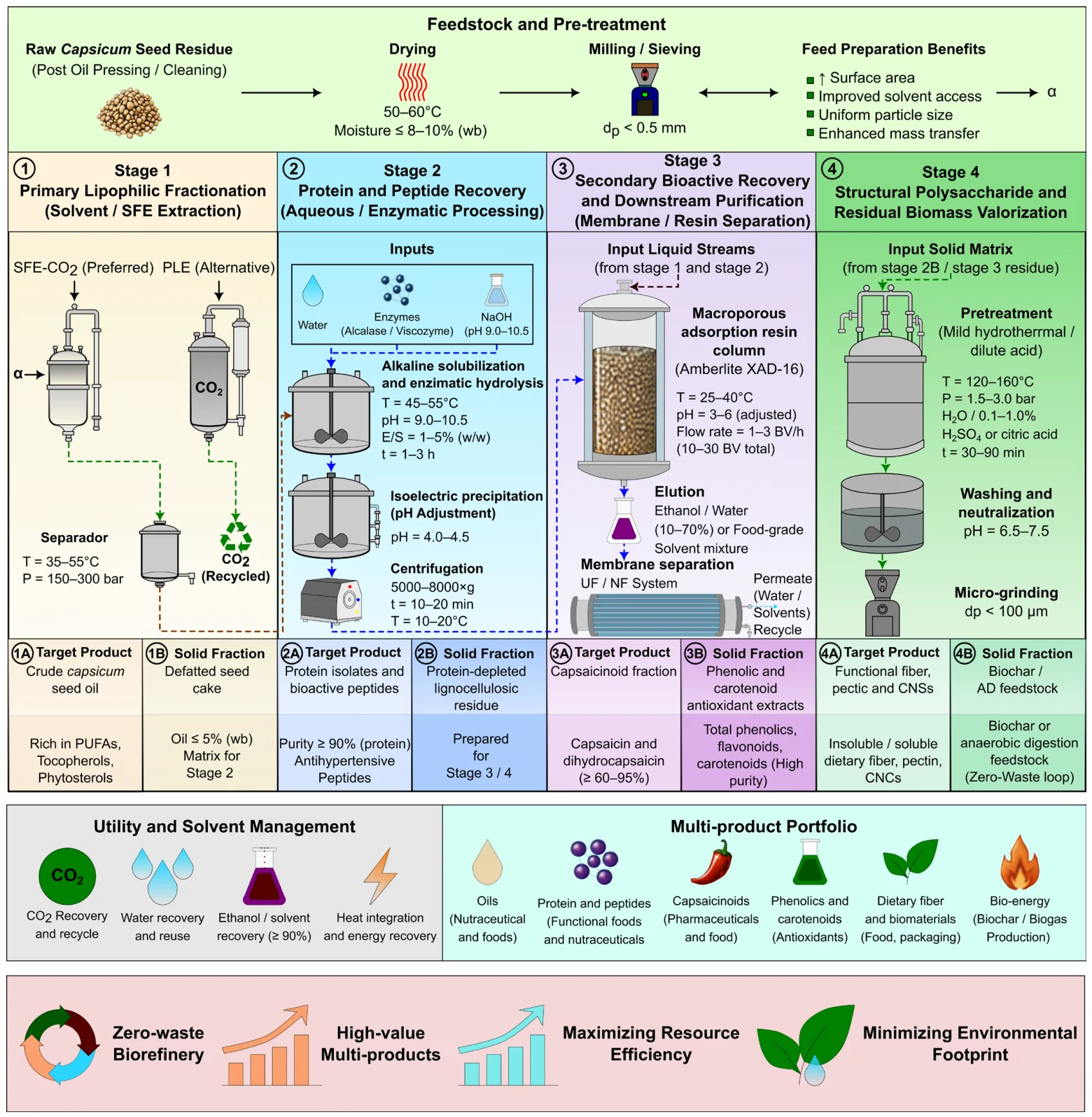
Integrated sequential cascade biorefinery pathway for the comprehensive valorization of *Capsicum* seed residues into a multi-product portfolio. The diagram illustrates the conceptual mass-flow conversion from feedstock conditioning (*dp* < 0.5 mm), where sub-graphs A and B denote the target bioactive fractions and the corresponding solid residues for stages 1–4, respectively. This is followed by four interconnected stages: (1) primary lipophilic extraction (SFE − CO_2_/PLE), (2) aqueous-enzymatic extraction of proteins and peptides from the defatted cake, (3) downstream purification of liquid streams using macroporous resins and membranes (UF/NF), and (4) hydrothermal and acid fractionation of the remaining lignocellulosic matrix. Closed-loop recovery of CO_2_, water, and solvents is proposed as a process-integration strategy to reduce resource consumption and residual waste generation; however, the corresponding recycling efficiencies require experimental validation at pilot and industrial scales.

Figure 3 illustrates a four-stage conceptual cascade pathway for *Capsicum* seed valorization. Based on operating conditions reported for individual unit operations, feedstock conditioning may involve drying at 50–60 °C to approximately 8–10% moisture, followed by milling to <0.5 mm. Stage 1 considers supercritical CO_2_ extraction for lipid recovery, whereas the defatted cake is subsequently directed to alkaline/enzymatic protein extraction at 45–55 °C and pH 9.0–10.5, followed by isoelectric precipitation at pH 4.0–4.5. Downstream liquid streams may then be treated using macroporous resins and membrane separation, for which capsaicinoid recoveries of up to 95.5% have been reported in individual studies [134]. The remaining lignocellulosic fraction is proposed for further valorization into fiber, biopolymers, biochar, or biogas. This configuration represents a conceptual integration of unit operations that have been investigated separately rather than a fully validated industrial cascade. Quantitative values associated with individual stages correspond to experimental results reported for specific extraction, fractionation, or valorization operations and should not be interpreted as the overall performance of the complete sequence. The proposed links between oil recovery, protein fractionation, bioactive purification, and residual biomass valorization are based on their technological compatibility. Accordingly, solvent recycling, stream integration, and final resource recovery remain prospective design elements that require validation through continuous pilot-scale operation, integrated mass balances, and subsequent techno-economic and environmental assessment.

### 7.9. Product Portfolio and Value Creation

The economic potential of *Capsicum* seed residues depends not only on the quantity of compounds recovered but also on the ability to transform individual fractions into products with defined technological or biological functions. Experimental characterization of *Capsicum annuum* seeds led to the identification of 45 phytochemical compounds; the resulting extract exhibited a total phenolic content of 193.7 mg GAE/g dry weight (DW) and a flavonoid content of 25.1 mg QE/g DW, as well as DPPH radical scavenging capacity with an IC_50_ of 45.0 μg/mL, supporting its potential as a source of antioxidant ingredients [59]. Accordingly, valorization strategies must link chemical recovery to specific application pathways (Table 4), evaluating each fraction by its functionality and market potential rather than extraction yield alone [135]. Accordingly, cascade biorefineries should incorporate downstream concentration and stabilization strategies into their product-development pathways, particularly when extracts are intended to become standardized ingredients rather than intermediate laboratory fractions [136].

To provide a consistent assessment of the valorization routes summarized in Table 4, valorization level was classified as Low, Medium, or High according to the degree of transformation and the proximity of the resulting product to practical application. Low was assigned to routes mainly limited to experimental treatment or preliminary recovery of valuable compounds. Medium was used for processes producing functional fractions or ingredients with demonstrated technological potential but requiring further validation before broader application. High was assigned to routes generating directly usable products, food ingredients, extracts, oils, or other value-added materials with demonstrated functionality and a clearly defined application.

## 8. Sustainability, Process Intensification, and Industrial Scalability

### 8.1. Extraction Yield Versus Overall Resource Recovery

Extraction yield alone is insufficient to evaluate the sustainability of *Capsicum* seed valorization because maximizing recovery of one fraction does not necessarily maximize the utilization of the original biomass. This distinction is evident in aqueous oleosome processing, where laboratory-scale separation recovered 66.96 wt% oleosomes, yet the process generated 31.4 GJ of total exergy loss, approximately twice that of the conventional oil-processing route [16]. Importantly, replacing laboratory assumptions with industrial material efficiencies reduced the estimated exergy loss to nearly one quarter of the original value, demonstrating that resource recovery depends strongly on losses occurring throughout the process rather than on extraction yield alone. Similarly, industrial *Capsicum* residue still contained 27.65 mg/100 g vitamin C after primary processing, while its 8.09% pectin yield illustrates that residual resources can remain available after the principal extraction step [10].

### 8.2. Energy Consumption and Energy Efficiency

Process intensification technologies can improve recovery efficiency, but their sustainability depends on how much energy is required to obtain a given quantity of valuable product. Research evaluating PEF treatment in red bell pepper demonstrates that increasing electrical energy changes structural characteristics, bioactive compounds, and quality attributes, highlighting the trade-off between treatment intensity and processing benefit. For *Capsicum* seed residues, this relationship means that energy demand should be assessed against the incremental recovery achieved after pretreatment or extraction. A technology that produces only a marginal improvement in recovery while substantially increasing energy consumption may be less attractive than a lower-intensity alternative integrated into a sequential process. Therefore, energy efficiency should be expressed in relation to recovered value rather than simply total energy consumption. This perspective is particularly important when scaling intensified extraction technologies from laboratory conditions toward continuous industrial processing [95].

### 8.3. Solvent Consumption, Toxicity, and Recovery

Solvent selection in *Capsicum* seed-residue valorization should consider not only extraction performance but also solvent consumption, toxicity, and the feasibility of downstream recovery. Water-based enzyme-assisted extraction of habanero seed residues demonstrated that bioactive compounds could be recovered using water as the extraction medium, with the process producing a 73.5% yield increment under optimized enzymatic conditions, thereby avoiding dependence on large volumes of organic solvents [75]. More recent solvent intensification has shifted toward natural deep eutectic solvents (NADESs), where enzyme-assisted extraction of capsaicin from *Capsicum* biomass achieved 9.91 ± 0.13 mg/g using a choline chloride–butylene glycol system containing 17.8% water, while maintaining satisfactory performance after 50-fold scale-up [97]. In contrast, conventional ethanolic extraction requires subsequent solvent removal, making solvent recycling essential for reducing material losses and process burdens [97]. Therefore, solvent sustainability should be assessed through the combined criteria of toxicity, solvent-to-solid ratio, recyclability, and compatibility with food-grade downstream processing.

### 8.4. Preservation of Bioactive Compounds and Product Quality

Drying is a critical conditioning step because moisture removal can improve material stability while simultaneously affecting bioactive retention and product quality. Experimental evaluation of three *Capsicum annuum* varieties showed that drying strategy influenced nutrient and aroma characteristics, with variable-temperature drying reducing losses of total sugars, acids, fat, and capsaicin compared with conventional thermal treatments [147]. Similarly, recent experimentation with *Capsicum chinense* demonstrated that drying technology markedly affected phenolic retention, antioxidant activity, and vitamin content; microwave drying at 300 W retained 317.22 mg GAE/g dry matter of total phenolics and 317.56 mg/100 g dry matter of vitamin C in red peppers [25]. In addition, analysis of 19 accessions from four *Capsicum* species demonstrated species- and cultivar-dependent changes in volatile composition after drying [70]. Integrating drying kinetics with bioactivity measurements provides a more meaningful basis for process optimization and helps ensure that technological intensification does not compromise the quality of the final valorized fraction [148]. Drying efficiency allows the initial conditioning step to support—rather than compromise—the performance of the entire valorization chain by being incorporated into the overall process design framework [147].

### 8.5. Process Integration and Reduction of Waste Streams

Process integration is essential for reducing residual streams because the sustainability of *Capsicum* seed biorefineries depends on the destination of intermediate fractions rather than on the efficiency of individual extraction steps. Experimental processing of pepper-derived residues demonstrated this principle by combining primary extraction with subsequent chromatographic fractionation, transforming an initial waste stream into multiple products while generating a less pungent residual oil instead of directing the extracted fraction to disposal [113]. For seed-specific residues, the same strategy can be extended by converting press-cake into food formulations, as demonstrated by the successful incorporation of roasted pepper seed press-cake into sauces, where roasting at 140–170 °C produced formulations with improved overall suitability and storage stability [130].

### 8.6. Techno-Economic Considerations

Techno-economic feasibility of *Capsicum* seed biorefineries depends on the interaction between processing scale, equipment requirements, operating costs, and the value assigned to sequentially recovered fractions. Economic assessment of sequential supercritical fluid and pressurized liquid extraction of *Capsicum chinense* showed that increasing extractor volume from laboratory to 500 L reduced the cost of manufacturing, while solvent-to-feed ratios of 4.9 and 10 were identified as favorable for capsiate and phenolic fractions, respectively [149]. More recent resource assessment of *Capsicum* residues similarly indicates that dedicated re-extraction of secondary compounds can remain economically constrained by high operational costs and limited yields, emphasizing the importance of integrating several products within the same process rather than relying on a single extract [10]. In seed processing, exergy analysis further demonstrated that replacing laboratory-scale material efficiencies with industrial efficiencies reduced total process exergy loss from 31.4 to 23.5 GJ, highlighting the economic relevance of minimizing material losses during scale-up [16].

### 8.7. Life-Cycle and Environmental Assessment

Life-cycle assessment (LCA) provides a useful basis for determining whether the recovery of *Capsicum* seed residues can translate into measurable environmental advantages. This consideration is relevant because pretreatment, drying, extraction, solvent recovery, and subsequent separation may increase energy and resource requirements. In *Capsicum* pubescens production, the most favorable scenario showed an impact of 0.092 kg CO_2_-eq per functional unit and achieved the lowest impact in five of six assessed categories, illustrating the influence of production conditions on environmental performance [141]. At the residue-management stage, pepper-processing biomass has shown a methane potential of 401 L/kg dry matter, indicating that fractions remaining after recovery of valuable compounds may retain energy potential [131]. Moreover, integrating pepper residues into anaerobic co-digestion increased electricity generation by 70% and economic benefits by 5.5-fold [150].

### 8.8. Scale-Up Challenges and Industrial Implementation

Industrializing separation processes for *Capsicum* seed residues requires balancing yield, exergy performance, and matrix complexity. For aqueous oleosome extraction, initial laboratory models exhibited high exergy losses (31.4 GJ), nearly double those of conventional solvent processing; however, applying industrial material efficiencies cut estimated exergy dissipation by 75%, demonstrating that process scaling significantly improves thermodynamic performance [16]. Similarly, scaling eco-friendly approaches—such as aqueous cellulase-assisted extraction (337.96 mg GAE/L phenolics [75]—depends on mitigating downstream water consumption and purification bottlenecks. Industrial implementation must therefore prioritize thermodynamic efficiency and process integration over single-parameter yield optimization.

## 9. Applications and Future Perspectives

The applications of *Capsicum* seed residues are closely determined by the separation technology used and the composition of the recovered fraction. Cold pressing mainly produces lipid-rich fractions suitable for food and cosmetic applications [119,151,152], whereas hydro-methanolic and enzyme-assisted extraction generate phenolic- and capsaicinoid-rich extracts with potential nutraceutical and health-related uses [59,60,75]. Aqueous oleosome extraction preserves intact lipid structures that can be incorporated into emulsions and bio-based materials [16,153]. Likewise, pressure-assisted defatting and protein/fiber fractionation provide functional macromolecular fractions that can be further processed into biodegradable films and other biomaterial systems [74,154]. Thus, the end use of each fraction depends directly on the selectivity and severity of the preceding separation step.

### 9.1. Food and Functional Ingredients

*Capsicum* seed residues offer opportunities for developing food and functional ingredients through the recovery of edible oils and bioactive-rich extracts. Cold-pressed sweet pepper seed oil obtained from *Capsicum annuum* by-products showed a smoke point of 230 °C when seeds were rapidly dried, supporting its potential for cooking and processed-food applications; consumer evaluation also indicated favorable organoleptic acceptance [151]. In another experimental study, hydro-methanolic extracts from *Capsicum annuum* seeds contained 193.7 mg GAE/g dry weight of phenolics and 25.1 mg QE/g of flavonoids, with DPPH, reducing-power, and chelating activities showing IC_50_ values of 45.0, 61.3, and 79.0 μg/mL, respectively [59]. Enzyme-assisted processing provides an additional route, as cellulase treatment of jalapeño seeds generated phenolic–carbohydrate aggregates with an average particle size of 660.9 nm and demonstrated immunostimulatory activity of 234.25% under optimized conditions [60].

### 9.2. Nutraceutical Applications

The bioactive profile of *Capsicum* seed residues supports their application in nutraceutical formulations. Enzymatic extraction of *Capsicum chinense* seeds yielded extracts rich in phenolics and capsaicinoids that suppressed nitric oxide production in RAW 264.7 macrophages down to 22.56%, confirming strong anti-inflammatory properties [75]. Similarly, hydro-methanolic profiling of Capsicum annuum seeds identified 45 phytochemicals—including phenolics, capsianosides, fatty acids, and sphingolipids—with potent DPPH radical scavenging activity ($IC_{50}\;=\;45.0\;\mu g/mL$) [59].

### 9.3. Antioxidant, Antimicrobial, and Health-Related Applications

Selective extraction of *Capsicum* seed residues yields protein- and metabolite-rich fractions with multi-target biological activities. A protein-enriched fraction (PEF3) from *Capsicum chinense* seeds induced 83.7% viability loss in *Fusarium oxysporum* by triggering reactive oxygen species accumulation, mitochondrial membrane dissipation, and caspase activation [152]. Similarly, ethanolic and hexane seed extracts demonstrated potent activity against *Candida glabrata* and *Candida tropicalis*, inhibiting biofilm formation, reducing fungal hemolytic activity, and suppressing intracellular Toxoplasma gondii proliferation at non-cytotoxic levels [153].

### 9.4. Cosmetic and Non-Food Applications

The recovery of lipid-rich fractions from *Capsicum* seed residues also creates opportunities for cosmetic and other non-food applications, particularly when separation processes are designed to preserve the functional characteristics of the recovered oil. Sweet pepper seed oil obtained from discarded seeds contained 86.5% unsaturated fatty acids, with linoleic acid representing 69.4%, while thermal and spectroscopic analyses indicated the absence of trans fatty acids and peroxides, supporting its potential as a stable lipid raw material for non-food formulations [119]. More direct evidence has been provided by experimental soap formulations incorporating *Capsicum annuum* seed oil, in which blends containing up to 10% seed oil showed satisfactory physicochemical characteristics and consumer acceptance, demonstrating the feasibility of converting a seed-processing residue into a personal-care ingredient [154]. In parallel, aqueous oleosome extraction has demonstrated the possibility of recovering intact oil-rich structures from *Capsicum* seeds, opening future opportunities for milder, solvent-reduced routes toward cosmetic emulsions and other oleochemical products [16].

### 9.5. Biomaterials and Other Emerging Applications

Beyond food, nutraceutical, and cosmetic uses*, Capsicum* seed residues provide protein-, fiber-, and lipid-rich fractions that can be incorporated into emerging biomaterial systems. Protein and crude fiber recovered from cold-pressed chili pepper seed oil by-products have been successfully converted into antimicrobial films, with the protein film reaching a tensile strength of 0.46 MPa and an elongation at break of 33.76%, while both protein-based and protein–fiber films inhibited *Staphylococcus aureus* and *Escherichia coli* [155]. In parallel, oleosomes isolated from chili seeds have been incorporated into soy-protein films, demonstrating the potential of seed-derived lipid structures to modify film barrier, mechanical, antioxidant, and antimicrobial properties [156]. These applications are supported by the efficient recovery of functional protein fractions from pepper seed meal, for which pressure-assisted defatting enhanced solubility, water- and oil-holding capacity, emulsification, and foaming properties compared with conventional defatting [74]. This suggests that cascade separation could transform residual seed fractions into biodegradable films, oleosome-based materials, and other bio-based platforms.

### 9.6. Food-Grade and Regulatory Considerations

The transition of *Capsicum* seed residues into food-grade ingredients requires control of microbial safety, physicochemical quality, oxidative stability, and compositional consistency throughout the recovery process. Recent experimental evidence demonstrated that γ-irradiation of *Capsicum annuum* seeds at 5.0 kGy reduced bacterial and fungal populations to below detectable levels while maintaining relatively limited changes in the quality of the resulting cold-pressed oil; however, increasing the irradiation dose progressively increased free fatty acids and peroxide values and decreased antioxidant activity [13]. Complementary characterization of sweet pepper seed oil showed a high proportion of unsaturated fatty acids and no detectable peroxides or trans fatty acids, supporting its suitability as a potential edible oil source [119]. In addition, green extraction of oil from red pepper seed waste and subsequent accelerated storage demonstrated that extraction technology significantly influences oxidative stability, emphasizing the need to control both processing and storage conditions when developing food-grade fractions [68]. Accordingly, future cascade biorefineries should integrate validated decontamination, oxidation control, compositional standardization, traceability, and application-specific safety assessment as prerequisites for regulatory acceptance of seed-derived ingredients.

### 9.7. Research Gaps and Future Technological Opportunities

Despite the increasing evidence on the valorization of *Capsicum* seed residues, an important research gap concerns the transition from isolated extraction or modification processes toward integrated and multifunctional biorefinery platforms. Recent studies demonstrate that ultrasound can substantially modify red pepper seed protein structure and improve solubility, emulsifying, foaming, and water- and oil-holding properties, while high hydrostatic pressure has produced further improvements in the functional performance of pepper seed protein isolates [83,127]. More advanced opportunities are emerging from the combination of recovered seed proteins with high-intensity separation and formulation technologies: ultrasonic homogenization has enabled the production of pepper seed protein nanoemulsions with controlled droplet size and stability under different environmental conditions, while enzymatic hydrolysis of red pepper seed proteins has opened routes toward bioactive peptide fractions with enhanced functionality [57,133].

The most critical research gap is the limited validation of integrated cascade processes beyond laboratory scale. Priority should therefore be given to demonstrating sequential recovery schemes under pilot conditions, with particular emphasis on preserving the functionality of downstream fractions, reducing solvent and energy consumption, and quantifying process yields across the complete biorefinery sequence. A second priority is the application of techno-economic analysis and life-cycle assessment to determine whether improved recovery and product diversification translate into viable and environmentally advantageous processes. Once these aspects are established, broader opportunities can focus on advanced formulation, membrane integration, enzymatic fractionation, digital monitoring, and data-driven process control. These emerging tools may further improve efficiency, but their implementation should follow the resolution of the more fundamental scale-up, integration, and sustainability gaps.

### 9.8. From Laboratory Extraction to Integrated Industrial Biorefineries

The transition of *Capsicum* seed-residue valorization from laboratory extraction to integrated industrial biorefineries remains constrained by the limited validation of sequential processes under pilot and industrial conditions. An integrated experimental approach applied to pepper-processing residues demonstrated that sequential extraction and chromatographic separation could generate multiple value-added streams, illustrating the feasibility of coupling recovery operations rather than treating each compound independently [113]. For seed-derived fractions, recent studies have increasingly adopted multivariable optimization, such as response surface methodology for the simultaneous adjustment of protein concentration, ultrasonic power, and treatment time, demonstrating the importance of considering interactions among operating parameters rather than optimizing individual variables separately [57].

Process integration should therefore prioritize sequential recovery of oil, protein, phenolic, peptide, and residual structural fractions while incorporating solvent and water recirculation, energy integration, and intermediate-stream characterization. At the industrial level, techno-economic analysis (TEA) and life-cycle assessment (LCA) will be essential to determine whether improved recovery yields translate into economically viable and environmentally preferable processes. Finally, digitalization, online sensors, process analytical technologies, and data-driven monitoring could support real-time control of extraction, separation, and product quality, enabling adaptive operation and more consistent performance during scale-up.

## 10. Conclusions

*Capsicum* seed residues represent a promising biomass resource because they contain recoverable oils, proteins, phenolic compounds, capsaicinoids, peptides, and other functional fractions. The reviewed evidence demonstrates the technical feasibility of recovering and fractionating these components through a range of extraction and downstream separation strategies. Collectively, the studies show that process performance depends strongly on the target fraction, matrix characteristics, operating conditions, and the degree of selectivity required.

The evidence does not support the identification of a single extraction or separation technology as universally superior. Instead, different operations provide complementary advantages depending on whether the objective is bulk recovery, selective enrichment, or purification of specific compounds. This supports the potential value of sequential processing, in which different fractions are recovered from the same residue and subsequently subjected to appropriate separation or purification steps. However, this cascade configuration remains a proposed process concept rather than a fully demonstrated industrial biorefinery.

Important gaps remain in the translation of individual laboratory-scale results into integrated processes. Differences in experimental conditions, scales, solvents, response variables, and reporting criteria currently limit direct comparison of technologies in terms of energy demand, solvent consumption, scalability, economic performance, and environmental impact. Therefore, the demonstrated feasibility of individual operations should not be interpreted as evidence of an already validated integrated biorefinery.

Future research should focus on integrated and sequential process schemes, supported by standardized mass balances, product-quality assessment, process stability, and pilot-scale validation. Techno-economic and life-cycle assessments should accompany process development to determine whether the proposed configurations can achieve efficient resource utilization and generate economically and environmentally viable value-added products from *Capsicum* residues.

## Figures and Tables

**Table 1:** Chemical composition and bioactive potential of *Capsicum* seed residues and associated fruit fractions reported in the literature.

Capsicum Species/Cultivar	Material/Fraction	Main Constituents	Quantitative Data	Bioactive Compounds	Biorefinery Relevance	Ref.
*C. annuum* var. *palmera*	Pericarp	Tocopherols, sterols, triterpenes, fatty acids	181 g extract; 38.7 g CH_2_Cl_2_; 28.8 g n-BuOH	α-Tocopherol, β-sitosterol, α/β-amyrin, lupeol, oleic acid, pyrrole-lactone	Bioactives	[30]
*C. annuum* var. *grossum*	Aerial parts; CHCl_3_ fraction	Phytosterols	β-sitosterol: 0.492 mg/mL	β-Sitosterol	Phytosterol recovery	[31]
*C. annuum* (8 cultivars)	Fruit + placenta + seeds	CPS, DCPS, PPH	PS: 2.1–124.2 mg/g; DCPS: 5.1–151.3 mg/g; PPH: 3.53–25.9 mg GAE/g; SHU: 32,840–1,986,880	Capsaicinoids, polyphenols, carotenoids	Bioactives	[32]
*C. chinense*, *C. annuum*, *C. baccatum*; 19 varieties	Chili pepper whole fruit, aqueous/EtOH extracts	Phenolic acids, flavonoids, tannins, capsaicinoids	23 compounds (H_2_O), 29 (EtOH); EtOH: 8 varieties >70% AChE/GLU inhibition; IC_50_: GLU 0.56 mg/mL, BChE 0.88 mg/mL, TYR 0.63 mg/mL	Capsaicin, dihydrocapsaicin, nordihydrocapsaicin, rutin, quercetin, naringenin, luteolin, apigenin	Recovery of antioxidant	[33]
*C. frutescens*, *C. annuum*	Whole fruit	Protein, fiber, phenolics, flavonoids, saponins, minerals	Protein: 1.52–1.88%; fiber: 5.18–5.72%; TPC: 5.36–18.85 μg GAE/mg	Phenolics, flavonoids, saponins	Functional ingredients; bioactive extracts	[34]
*C. annuum* L. (Byadagi, Guntur, Cherry)	Seeds and whole fruit	Oleoresins, capsaicinoids, carotenoid pigments	Seed extract yield: 2.64–7.69%; capsaicin: 0.12–1.61%	Capsaicin, carotenoids	Recovery of oleoresins and capsaicinoids	[35]
*C. chinense* (29 Habanero hybrids)	Dried whole fruits	Capsaicinoids	Total: 27.33–85.05 mg/g DW; capsaicin: 19.95–73.55 mg/g DW; DHC: 4.57–14.36 mg/g DW	Capsaicin, dihydrocapsaicin	Recovery of high-pungency capsaicinoids for food	[36]
*Capsicum* spp. (BRPS, LPS, WPS)	Chopped pepper seeds	Carbohydrates, proteins, lipids, fatty acids	Carbohydrates: 55.42–67.92%; protein: 12.96–16.09%; fat: 13.07–17.29%; unsaturated FA: 79.50–83.36%; linoleic acid: 69.14–75.22%	Linoleic acid, essential amino acids	Recovery of seed oil, protein and fiber-rich flour	[37]
*C. annuum*, *C. chinense*, *C. baccatum* (123 genotypes)	Whole pepper fruits	Capsaicinoids	Total SHU: ~50–1.45 million; mean: 86,894 SHU	Capsaicin, dihydrocapsaicin, nordihydrocapsaicin	Selective genetic improvement and the recovery of bioactive compounds	[38]
*C. chinense* (19 accessions)	Seedless ripe fruits	Phenolics, carotenoids, anthocyanins	Phenolics: 113.12–233.58 mg CAE/100 g; carotenoids: up to 195 mg β-carotene/100 g	Phenolic compounds, carotenoids, anthocyanins	Selection of bioactive-rich genotypes	[39]
*C. annuum* (27 cultivars)	Fruit extracts	Alcohols, hydrocarbons, esters, ketones	71 volatile compounds; alcohols 26.13%, hydrocarbons 18.82%, esters 14.97%	Volatile compounds with cytotoxic and antimicrobial activity	Recovery of volatile fractions for functional food applications	[40]
*C. annuum* (green, yellow, red bell pepper)	Seeds and pulp	Phenolics, flavonoids, tannin	Seed TPC: 0.43–0.53 mg GAE/g FW; TFC: 0.54–4.98 μg QE/g FW; 59 phenolics identified	Phenolic acids, flavonoids, polyphenols, lignans	Recovery of antioxidant phenolic compounds from seed residues.	[41]
*C. annuum* (DLJ, ZDT, XML, CTJ)	Dried fruits, pericarp + seeds	Volatiles, lipids, phenolics, capsaicinoids	452 volatiles; 53 non-volatiles; 64 capsaicinoid nodes	Capsaicin, dihydrocapsaicin, nonivamide	Recovery of aroma compounds, capsaicinoids, phenolics and lipids	[42]
*C. annuum* L. (paprika)	Paprika oleoresin; unsaponifiable fraction	Phytosterols, carotenoids	β-sitosterol: 300 mg (15%); β-citraurin: 200 mg (22%); purity of β-citraurin: 88%	β-Sitosterol, β-citraurin	Purification of phytosterols and carotenoids from paprika oleoresin	[43]
*C. annuum* L. (sweet pepper)	Seeds; seed oil	Fatty acids, fatty acid esters, phenolic compounds	Oil yield: 14.7%; DPPH inhibition: 74%; peroxide value: 12.27 mEq/kg at 2.5% SPSO	Cis-palmitoleic acid, myristic acid, pentadecylic acid	Recovery of seed oil as a lipid-rich fraction	[44]
*C. annuum* (12 cultivars; red, yellow, green)	Dry plant material/fruits	Phenolic acids, flavonoids, polyphenols	Extract yield: 20.21–39.41 g/100 g; TPC: 30.51–60.81 mg GAE/g; TFC: 9.10–30.91 mg CE/g; gallic acid: 54.72–1058.72 mg/kg; catechin: 83.47–691.79 mg/kg	Gallic acid, catechin, phenolic acids, flavonoids	Recovery of polyphenol-rich extracts	[45]
*C. annuum*	Seeds	Capsaicinoids, fatty acids, volatiles	Linoleic acid predominant; PUFA unchanged; capsaicinoids ↓ with temperature	Capsaicin, DHC, linoleic acid	Roasted functional ingredient	[46]
*C. annuum* (green, orange, red)	Fruits, stalks, seeds	Carbohydrates, proteins, phenolics, fatty acids	22 phenolics; 34 volatiles	Phenolics, PUFA	Recovery of antioxidants and antimicrobials	[47]
*C. annuum* (‘Jalapeño’, ‘Baklouti’, ‘Black Prince’, ‘Black Cobra’)	Fruits	Phenolics, capsaicinoids	DPPH IC_50_: 2.09 mg/mL; IC_100_: 3.84 mg/mL; anti-inflammatory >80%	Catechin, capsaicin, DHC, vanillic, syringic and ferulic acids	Antioxidant and anti-inflammatory extracts	[48]
*C. annuum* (green, yellow, red)	Fruits	Phenolics, flavonoids	TPC: 15.55–30.15 mg GAE/g; TFC: 5.11–13.04 mg CE/g; DPPH: 18.31–72.76%	Tannic, chlorogenic, caffeic, salicylic acids; quercetin	Antioxidant and antimicrobial extracts	[49]
*C. annuum* L. (Baklouti, Paprika)	Dried pepper powder	Sesquiterpenes, fatty acids, sugars	31 VOCs; sesquiterpenes: 65.4%; α-curcumene: 16.0–19.7%; β-sesquiphelandrene: 13.8–14.7%; oleic acid: 46.8–54.5%; Paprika: linoleic acid 28.1%	Zingiberene, α-curcumene, β-caryophyllene, limonene	Volatile compounds, lipid fraction, and antimicrobial extracts	[50]
*C. chinense* Jacq. (Naga chilli)	Fresh fruit	Capsaicinoids, hydrocarbons, ketones	Nonadecane: 21.28%; 1-dimethyl(phenyl)silyloxypentane: 14.53%; capsaicin: 13.55%; 2-pentanone: 11.42%; capsaicin (by HPTLC): 0.833 mg/g fresh weight	Capsaicin and other capsaicinoids	Recovery of bioactive compounds with antimicrobial activity	[51]
*Capsicum* spp. (chili powder)	Powdered dried fruits; extract and frying oil	Carotenoids, capsaicinoids	Powder: total carotenoids 270.3 mg/kg; capsanthin 166.2 mg/kg; capsaicinoids 80.5 mg/kg. In oil at 130–190 °C: total carotenoids 13.3–60.0 mg/kg; carotenoid extraction 19.7–93.2%.	Capsanthin, capsorubin, capsaicin	Extraction of pigments and capsaicinoids using oil	[52]
*C. frutescens* L. (purple chili)	Powdered dried fruits; hydroethanolic extract	Capsaicinoids, phenolics, flavonoids, organic acids	Extract recovery: 45.65%; capsaicin: 4339.26 µg/g extract; DHC: 3529.15 µg/g; N-DHC: 838.92 µg/g; TPC: 11.92 mg GAE/g DW; TFC: 25.01 mg QE/g DW; ORAC: 607.63 µmol TE/g DW	Capsaicin, dihydrocapsaicin, nordihydrocapsaicin, fisetin	Obtaining extracts rich in capsaicinoids and phenolics	[53]

Note. ↓, decrease, AChE, acetylcholinesterase; BChE, butyrylcholinesterase; CAE, catechin acid equivalents; CE, catechin equivalents; DHC, dihydrocapsaicin; DPPH, 2,2-diphenyl-1-picrylhydrazyl; DW, dry weight; EtOH, ethanol; FA, fatty acids; FW, fresh weight; GAE, gallic acid equivalents; GLU, glucosidase; HPTLC, high-performance thin-layer chromatography; IC_50_, half-maximal inhibitory concentration; IC_100_, concentration required for 100% inhibition; N-DHC, nordihydrocapsaicin; PPH, phenolic phytochemicals; PUFA, polyunsaturated fatty acids; QE, quercetin equivalents; SHU, Scoville Heat Units; SPSO, sweet pepper seed oil; TE, Trolox equivalents; TFC, total flavonoid content; TPC, total phenolic content; TYR, tyrosinase; VOCs, volatile organic compounds.

**Table 2:** Operating conditions, extraction performance, target compounds, and limitations of technologies applied to *Capsicum* seed residues.

Technology	Capsicum Species/Cultivar	Feedstock/Pretreatment	Solvent/Extraction Medium	Main Operating Conditions	Extraction Performance	Target Compound/Fraction	Scalability Assessment	Ref.
Low-tech extraction	Red chili pepper	Red chili pepper	Hydroalcoholic solution/Soybean oil	1:10	<50/40–70	Capsaicinoids	High	[98]
Solvent/UAE	*Capsicum* spp.; 14 cultivars	Pulp, dried at 25 °C for 20 days	Ethanol	20 min; 20 kHz; 1:10	TPC 104.1–634.1 mg GAE/100 g; flavonoids up to 97 mg QE/100 g; capsaicin up to 922 mg/100 g	Phenolics, flavonoids, capsaicin	Low	[99]
Supercritical fluid extraction	*C*. *annuum*	Paprika, dried and milled	Supercritical CO_2_	NR	7.22–9.94	Carotenoids, capsanthin and fatty acids	High	[94]
Soxhlet extraction	*C*. *annuum* L., ‘Jalapeño’	Seeds; drying at 60 ± 2 °C, grinding, <595 μm	Petroleum ether (40–60 °C)	10 g; 6 h; vacuum evaporation at 60 °C	Oil characterized; iodine index 168.003 g I_2_/100 g	Seed oil	Low	[100]
Conventional extraction	*Capsicum* spp.	Freeze-dried green pepper, powdered to 80 mesh	Ethanol	1:10	10.22	Oleoresin and phenolic compounds	Low	[80]
Enzyme-assisted extraction (EAE)	*C*. *chinense*, Habanero	Seeds; enzymatic pretreatment	Water + cellulase	1:15 (*w*/*v*); 250–2500 UI/L; 30–60 °C; 30–150 min	Yield increase up to 73.5%; CAP up to 310.23 μg/mL	Phenolics, capsaicin (CAP), dihydrocapsaicin (DHC)	Low	[75]
Microwave-assisted extraction (MAE)	*Capsicum* spp.	Freeze-dried, 80-mesh powder	Ethanol	1:10	NR	Oleoresin and piperine/bioactive compounds	Low	[17]
Supercritical CO_2_ extraction	*C*. *annuum*	Red pepper, extruded/densified material	Supercritical CO_2_	NR	NR	Oleoresin and carotenoids	Medium	[101]
Green UAE	*C*. *chinense* Cumari do Pará	Pepper material; different maturity stages and locations	Soybean, Brazil-nut or palm-oil	NR	NR	Capsaicin, carotenoids and phenolics	Medium	[102]
Ultrasound-assisted extraction (UAE)	*Capsicum* spp./chili pepper	Ground, <1 mm	Water/70% ethanol	1:10; 30 kHz; 100 W; 10–30 min	TPC; UV profile improved	Phenolics	Low	[103]
Microwave-assisted extraction	*C*. *annuum* var. *glabriusculum*	Chiltepin pepper, dried/powdered	Hydroalcoholic solvent	Optimized by RSM	NR	Bioactive compounds and phenolics	Low	[90]
UAE/NADES	*C*. *chinense* ‘Jaguar’	Leaves; freeze-/oven-dried, ground < 500 µm	ChCl:Fru or MA:Fru NADES:water (50:50)	42 kHz, 135 W; 10–30 min; 1:12	TPC up to 36.18 mg GAE/g; selective polyphenol recovery	Catechin, chlorogenic acid, quercetin + luteolin	Medium	[104]
Pilot-scale supercritical CO_2_	*C*. *annuum* paprika	Dry paprika; pilot-scale extraction	Supercritical CO_2_	NR	NR	Oleoresin, carotenoids and volatile compounds	High	[93]
Ultrasound-assisted extraction (UAE)	*Capsicum annuum* L., Xkatik/Xcatik	Seeds, pericarp, placenta; fresh/dried, 60 °C, 24 h, 500 µm	Ethanol/water 70:30 (*v*/*v*)	20 kHz, 90% amplitude, 10 min; 2 g/20 mL	TPC 2.71–15.94 mg GAE/g DW; TFC up to 7.59 mg QE/g DW	Polyphenols, flavonoids, capsaicinoids	Low	[105]
UAE/maceration/Soxhlet/SFE/SFEC	*C. chinense* Habanero	Red Habanero waste	Organic solvents/supercritical CO_2_	NR	NR	Capsaicinoids and carotenoids	Low	[29]
Supercritical CO_2_	*C. annuum* Senise	Red pepper processing wastes	Supercritical CO_2_	NR	NR	Capsanthin-rich extracts	Medium	[106]
Ultrasound-assisted extraction (UAE)	*C. chinense*/Red Habanero	Seeds, peel, fiber, pulp; frozen −20 °C; 4–10 mm	Ethanol 96%; S/L 1:20	8–10 min; APD ≈ 1.0 W/mL; 26 kHz	Yield 26.54%; CAP 8.28 mg NVA/g; TPC 5.05 mg GAE/g; FRAP 134.40; DPPH 32.64	Capsicum oleoresin; capsaicinoids, phenolics, carotenoids	Medium	[107]
Surfactant-assisted solvent extraction	*C. annuum*/green chili	Dried, shade-dried 10 d; fine powder	Ethyl acetate + Tween-80	6 h; RT; shaking; S/L 1:30; Tween-80 > CMC	72.74 mg/10 g (ethyl acetate + Tween-80); up to 61.6 mg/10 g in acetone + Tween-80	Capsaicin	Low	[108]
Green seed-oil extraction	*C. annuum*	Red pepper seed waste	Vegetable oil/green extraction medium	NR	NR	Seed oil and fatty acids	Medium	[79]
Ultrasound-assisted extraction (UAE)	*C. chinense* (Bode, Habanero); *C. frutescens* (Malagueta)	Freeze-dried and ground whole fruits	Hydroethanolic solvent (30:70 *v*/*v*)	1:50 (*w*/*v*); 40 kHz; 280 W; 560 W/L; 1 h; ~40 °C; 5 °C/48 h static pre-extraction	UAE increased total capsaicinoid extraction vs. non-UAE	Capsaicin, dihydrocapsaicin	Low	[109]
Oil-assisted extraction	*C. frutescens* Hiyung	Chili pepper material infused in carrier oil	Palm olein/coconut oil	NR	NR	Capsaicinoid-rich chili oil	Medium	[110]
Ultrasound-assisted extraction (UAE)	*C. chinense* (Bode, Habanero); *C. frutescens* (Malagueta)	Freeze-dried, ground fruit	Ethanol/water 70:30	40 kHz, 280 W, 1 h, ≈40 °C	Higher capsaicinoid recovery than control	Capsaicinoids, phenolics	Low	[49]
Acid extraction	*C. annuum* green bell pepper	Dried pepper → ethanol/acetone washing → alcohol-insoluble residue	HCl, pH 1–3	5 g/100 mL	NR	Pectin-like polysaccharides and fiber-rich fraction	Medium	[111]

Note. →, followed by, Scalability assessment was classified according to the highest level of process demonstration reported in the corresponding study: Low = laboratory-scale experimentation without evidence of scale-up or process transfer; Medium = laboratory optimization accompanied by scale-up-oriented parameters, product validation, or partial evidence of process transfer; High = pilot-scale operation, demonstrated scale-up, industrially relevant processing, field application, or documented process transfer. Abbreviations: NR: No reported; CMC, critical micelle concentration; CO_2_, carbon dioxide; DHC, dihydrocapsaicin; EAE, enzyme-assisted extraction; FRAP, ferric reducing antioxidant power; GAE, gallic acid equivalents; MAE, microwave-assisted extraction; NADES, natural deep eutectic solvent; NVA, nordihydrocapsaicin equivalents; QE, quercetin equivalents; RSM, response surface methodology; S/L, solid-to-liquid ratio; SFE, supercritical fluid extraction; SFEC, supercritical fluid extraction with cosolvent; TFC, total flavonoid content; TPC, total phenolic content; UAE, ultrasound-assisted extraction.

**Table 3:** Advanced separation and fractionation strategies for recovering and purifying valuable compounds from *Capsicum* seed-derived streams.

Input Stream/Extract	Separation/Fractionation Technology	Separation Principle	Target Compound/Fraction	Operating Conditions	Recovery (%)	Purity/Concentration	Final fraction/Product	Potential Application	Scalability	Ref.
*C*. *annuum* fruit extract	Chloroform fractionation	Solvent partition	β-Sitosterol	Chloroform fraction	NR	0.492 mg/mL DW	β-Sitosterol-rich fraction	Nutraceutical; pharmaceutical	Low	[31]
Paprika *C*. *annuum*	SC-CO_2_/SC-CO_2_ + EtOH	Supercritical fluid	Carotenoids; PUFAs	50 °C; 45 MPa/40 °C; 25 MPa	NR	NR	Carotenoid-rich extract	Food; nutraceutical	High	[94]
*Capsicum* seed aqueous extract/oleosome-containing slurry	Aqueous oleosome extraction	Recovery of intact oil bodies from the aqueous seed matrix and separation from non-oil material	Oleosome-rich fraction	Aqueous extraction; process optimization reported by the authors	66.96 wt%; potentially up to 90 wt%	NR	Oleosome-rich fraction	Alternative lipid recovery and food applications	Medium	[16]
Habanero *Capsicum chinense* seeds	EAE + solid–liquid separation	Cellulase-mediated disruption of seed cell walls facilitates release of intracellular compounds	Phenolic- and capsaicinoid-rich extract	30–60 °C; 0–150 min; 250–2500 UI/L cellulase; 1:15 *w*/*v*	73.5% extraction-yield increment under 60 °C/2500 UI/L/150 min	TPC up to 337.96 mg GAE/L; CAP up to 310.23 μg/mL; DHC up to 167.72 μg/mL	Bioactive aqueous extract	Anti-inflammatory/nutraceutical applications	Low	[75]
Red pepper *C*. *annuum*	SC-CO_2_	SC-CO_2_	Carotenoids; oleoresin	60 °C; 50 MPa	Carotenoid-rich oleoresin	NR	NR	Food; antioxidant ingredient	High	[101]
*C. chinense*	SFE–CO_2_	SFE–CO_2_	Capsaicin; dihydrocapsaicin	45 °C; 1450 psi; 60 min	NR	37.09 ± 0.84; 10.17 ± 0.18 mg/g extract	Capsaicinoid-rich oleoresin	Food; pharmaceutical	High	[17]
*C. chinense* pepper	UAE + soybean oil	UAE	Carotenoids; capsaicin; phenolics	UAE + edible oil	69%	NR	Bioactive-enriched oil extract	Food; nutraceutical	Medium	[102]
Paprika oleoresin	Saponification + extraction + column chromatography	Hydrolysis of saponifiable components followed by selective chromatographic separation	β-Sitosterol and β-citraurin	Saponification, extraction and column chromatography	NR	Increased purity of isolated compounds	Isolated β-sitosterol and β-citraurin	Functional foods and pharmaceutical applications	Low	[43]
Hot pepper seed oil byproduct	Protein and fiber isolation/fractionation	Fractionation of cold-pressed seed-oil residue into protein- and fiber-rich fractions	Protein and dietary fiber	Cold-pressed HPOB followed by isolation of protein and fiber fractions	NR	Protein and fiber contents characterized	Protein- and fiber-rich fractions	Low-fat salad dressing and functional food formulations	Medium	[123]
Habanero leaves	UAE/NADES	Solvent–solute affinity; H-bonding	Catechin; chlorogenic acid; quercetin + luteolin	MAc; 10–20 min UAE; FzD/OvD	NR	51.14; 16.05; 5.37 mg/g	Selective polyphenol extracts	Food; cosmetic; pharmaceutical; nutraceutical	Medium	[104]
Chiltepin pepper	MAE	MAE	Phenolics; vitamin C; biocompounds	77.84 min; 360 mL H_2_O; 0.536 mm	NR	504.26 mg GAE/100 g; 3.48 mg/g	Bioactive extract	Biological; pharmaceutical	Low	[90]
Dry paprika	SC-CO_2_	SC-CO_2_	Carotenoids	45 MPa; 50 °C; 74 min	10.05%	4.21% carotenoids	Carotenoid-rich extract	Food; functional	High	[93]
Red bell pepper byproduct	Green extraction + extractive fractionation	Differential solubility of phenolics and polysaccharides in aqueous/ethanolic media	Phenolic and polysaccharide fractions	Water and 10–20% ethanol; 30, 50 and 90 °C; laboratory and Timatic^®^ extraction	Polysaccharides up to 10.4% (dry matter)	Phenolic content of dried extracts 8.0–11.2 mg/g	Phenolic-rich and polysaccharide-rich extracts	Functional ingredients and bioactive products	High	[124]
Xcatik pepper seeds	UAE	UAE	Phenolics; capsaicinoids	Fresh/dehydrated; UAE	NR	11.67 ± 3.34 mg GAE/g DW	Bioactive extract	Food; nutraceutical	Low	[105]
Red Habanero *Capsicum chinense* waste	Maceration/UAE/Soxhlet/SFE/SFEC	Differences in solvent polarity and supercritical-fluid selectivity generate extracts with different phytochemical profiles	Capsaicinoid- and carotenoid-rich fractions	Maceration, ultrasound-assisted maceration, Soxhlet, SFE and SFE with co-solvent	NR	Phytochemical profiles differed according to extraction process	Bioactive-rich extracts	Recovery of capsaicinoids and carotenoids	Low	[29]
Senise red pepper waste	SC-CO_2_	Selective solubilization of carotenoids under supercritical conditions	Capsanthin-rich fraction	Supercritical CO_2_; pressure and temperature varied	70.2% recovery relative to solvent extraction	Capsanthin-rich extract; quantitative concentration depends on optimum condition	Capsanthin-rich extract	Natural colorants and high-value ingredients	Medium	[106]
Red pepper seeds	Protein isolation + MN/US	Protein recovery; particle disruption	Protein isolate	MN 6 min; US 720 W, 40 kHz, 10 min	NR	NR	Functional protein isolate	Food ingredient	Medium	[125]
Red pepper seeds	UAE + ethanol extraction	Solvent extraction/UAE	Oil; phenolics; tocopherols	UAE + ethanol	16.80%	241.1 mg/kg phenolics; 1801.2 mg/kg tocopherols; 167.0 mg/kg phenolics	Seed oil	Food; nutraceutical	Medium	[79]
*Capsicum* pepper extract	UAE + microencapsulation	UAE enhances capsaicinoid release; subsequent encapsulation stabilizes extracts	Capsaicinoid/phenolic-rich extract	UAE followed by microencapsulation; extract concentrations evaluated	Higher capsaicinoid recovery than static extraction; exact value NR	Microcapsules containing 0.3 and 0.5% extract showed high phenolic content	Pepper-extract microcapsules	Food incorporation and functional ingredients	Medium	[109]
Paprika/oleoresin	Ethanol extraction + HPLC	Solvent extraction; chromatographic separation	Capsaicin	Ethanol; HPLC	97.22–101.96%	9.77–443.10 mg/100 g	Capsaicin-containing extract	Pungency/quality control	Low	[118]

Note. Scalability was classified as Low, Medium, or High based on the reported process scale and evidence of scale-up. Low refers to laboratory-scale procedures without demonstrated scale-up; Medium indicates technologies with feasible adaptation to larger-scale processing or partial application-oriented validation; and High denotes processes demonstrated at pilot scale or supported by established large-scale processing equipment. Abbreviations: DW: dry weight; SC-CO_2_: supercritical carbon dioxide; EtOH: ethanol; PUFAs: polyunsaturated fatty acids; NADES: natural deep eutectic solvent; UAE: ultrasound-assisted extraction; MAc: malic acid; FzD: freeze-drying; OvD: oven-drying; NR: not reported; MAE: microwave-assisted extraction; GAE: gallic acid equivalents; MN: micronization; US: ultrasound.

**Table 4:** Value-added products, applications, and valorization routes derived from *Capsicum* seed fractions.

Seed Fraction/Biomass Stream	Main Target Compounds	Valorization Route/Technology	Value-Added Product	Potential Application	Functional/Technological Value	Valorization Level	Ref.
Bang Chang chili pepper (*C. annuum var. acuminatum*)	Capsaicin; β-carotene; γ-oryzanol; α-tocopherol; phenolics	RBO maceration	*Capsicum* extract in rice bran oil	Food seasoning; external medicinal use	Antioxidant; anti-inflammatory; anti-diabetic; non-toxic; non-pungent	High	[137]
Hiyung chili (*C. frutescens L*.)	Total capsaicinoids	Oil infusion; 24/48/72 h	Chili oil	Food seasoning	Pungency; oxidative stability; sensory acceptance	High	[110]
Fresh green/red chili pepper	Capsaicinoids; volatile compounds	Co-milling with olives	Chili-flavored olive oil	Food seasoning	Increased capsaicinoids; fresh pepper aroma; improved sensory quality	High	[138]
Red sweet pepper powder	Bioactive compounds; antioxidants	Drying at 45 °C + grinding + Soxhlet extraction with 96% ethanol	Concentrated pepper extract	Sauces; meat products	Flavor; aroma; color; antioxidant functionality	High	[139]
Whole red pepper tissue	Carotenoids; vitamin C	PEF (1–10 kJ/kg)	PEF-treated pepper	Food processing	Permeabilization; enhanced bioactive release	Low	[140]
Rocoto biomass	Capsaicinoids	Organic LTG + biochar	High-pungency rocoto	Food	125,601 SHU; 0.092 kg CO_2_ eq/FU	Medium	[141]
*Capsicum* residue	Residual capsaicin	Anaerobic fermentation; composting	Pectin; feed; VFAs; compost	Food; feed; agriculture	462.09 mg/L VFAs; 3.16% VFAs/SCOD	Hight	[10]
Red pepper by-products	Carotenoids; phenolics	HHP, 600 MPa/5 min	Stabilized RP ingredient	Food	<1 log CFU/g; ΔE 0.4–0.7; AA stable	Hight	[142]
RP by-product	Phenolics; carotenoids; fiber	HPTT, 600 MPa/5 min	Stable food ingredient	Food	TPC, TCC, ABTS stable	Medium	[143]
Hot pepper seed oil by-product	Protein; fiber	P + F isolation	Low-fat salad dressing	Food	IP: 5.11–7.04 h; ζ: −34.70 to −46.70 mV	Hight	[123]
Dried pepper biomass	Phenolics; capsaicinoids	LPCP, 10–30 min	Decontaminated dried pepper	Food	TMAB: −0.37–1.41 log; TYAM: −0.70–2.68 log; ↑ bioaccessibility	Low	[144]
Sweet pepper seeds	Seed oil; fatty acids; phenolics	Oil extraction + GC-MS	SPSO	Food preservation	14.7% yield; 74% DPPH; PV: 12.27 mEq/kg	Hight	[44]
Red bell pepper by-products	Phenolics; polysaccharides	Green extraction; Timatic^®^	Phenolic/polysaccharide extracts	Food; nutraceutical	8.0–11.2 mg/g phenolics; 10.4% polysaccharides	Medium	[124]
Pepper seed by-products	Polyphenols; flavonoids; phenolic acids	S–L extraction; EtOH 20%	Phenolic extract	Food; nutraceutical	TPC: 6.7829 mg GAE/g DW; 115 metabolites	Hight	[145]
Red habanero chili waste	Capsaicinoids; carotenoids	SFEC	Bioactive extract	Food; nutraceutical; industrial	14.9 mg/g capsaicinoids; 292.09 µg/g carotenoids	Medium	[29]
Red pepper seeds	Protein	MN 6 min + US 10 min	RPSPI	Food ingredient	↑ solubility, emulsifying and foaming; ↑ ABTS/DPPH; ↑ digestibility	High	[125]
Red habanero by-product	Capsaicinoids; phenolics; carotenoids	UAE, 8 min/0.87 W/mL	Capsicum oleoresin	Food ingredient	26% yield; 7 mg/g capsaicinoids; 466,000 SHU; −95% energy	Medium	[107]
Red pepper seeds	Oil; linoleic acid; tocopherols; phenolics	Ethanol extraction/UAE	Seed oil	Food; nutraceutical	16.80% yield; 72.00% linoleic acid; 1801.2 mg/kg tocopherols	High	[79]
Pepper crop residues	Biomass	Thermochemical conversion	Biofuel	Heat/energy	15.73 MJ/kg NCV	Medium	[146]
Capsicum pepper biomass	Capsaicinoids; phenolics	UAE + microencapsulation	Bioactive microcapsules	Functional foods	↑ capsaicinoid recovery; high encapsulation efficiency	Low	[109]
Red chili pepper biomass	Phenolics; capsaicinoids	MeOH/H_2_O extraction	Bioactive extract	Food; nutraceutical	DPPH >50%; anti-inflammatory >80%	Low	[48]
Chili biomass	Carotenoids; capsaicinoids	Oil extraction, 130–150 °C	Chili oil	Food	>90% extraction; 63.0 mg/kg carotenoids; 20.9 mg/kg capsaicinoids	Medium	[52]

Note. Valorization level was classified as Low, Medium, or High. Low indicates preliminary or experimental valorization with limited evidence of practical application; Medium refers to functional products or fractions with demonstrated application potential but requiring further development; and High denotes directly applicable value-added products or ingredients with demonstrated functionality and a clearly identified end use. Abbreviations: ↑, increase; PO-48 (palm olein, 48 h); HHP = high hydrostatic pressure; RP = red pepper; AA = antioxidant activity; HPTT: high-pressure thermal treatment; TPC: total phenolic content; TCC: total carotenoid content; ABTS: antioxidant activity; P: protein; F: fiber; IP: induction period; ζ: zeta potential; LPCP: low-pressure cold plasma; TMAB: total mesophilic aerobic bacteria; TYAM: total yeast and mold; SPSO: sweet pepper seed oil; PV: peroxide value; S–L: solid–liquid; EtOH: ethanol; DW: dry weight; SFEC: supercritical fluid extraction with co-solvent; RPSPI: red pepper seed protein isolate; MN: micronization; US: ultrasound; UAE: ultrasound-assisted extraction; SHU: Scoville Heat Units; GAE: gallic acid equivalents; NCV: net calorific value; MeOH: methanol; H_2_O: water.

## Data Availability

No new data were created or analyzed in this study. Data sharing is not applicable to this article.

## Supplementary Materials

The following supporting information can be downloaded at: https://www.mdpi.com/article/10.3390/foods15203699/s1, Table S1: PRISMA 2020 Checklist. Reference [24] is cited in the supplementary materials.
