# Supplementary material for: Advanced Separation Technologies for Cascade Biorefinery Valorization of Capsicum Seed Residues
Source: Foods. 2026 Oct 17;15(20):3699. doi: 10.3390/foods15203699 (PMC13704395; doi:10.3390/foods15203699)
Supplement: Supplementary file 1 [file foods-15-03699-s001.zip › foods-4609032-supplementary.pdf]

PRISMA 2020 Checklist

Section and Topic	Item #	Checklist item	Location where item is reported
**TITLE**			
Title	1	Identify the report as a systematic review.	Page 1: Line 2 and 3
**ABSTRACT**			
Abstract	2	See the PRISMA 2020 for Abstracts checklist.	Page 1: Line 18 - 37
**INTRODUCTION**			
Rationale	3	Describe the rationale for the review in the context of existing knowledge.	Page 2, 3 and 4: Line 85 - 107
Objectives	4	Provide an explicit statement of the objective(s) or question(s) the review addresses.	Page 4: Line 109 – 114
**METHODS**			
Eligibility criteria	5	Specify the inclusion and exclusion criteria for the review and how studies were grouped for the syntheses.	Page 4: Line 129 – 143
Information sources	6	Specify all databases, registers, websites, organisations, reference lists and other sources searched or consulted to identify studies. Specify the date when each source was last searched or consulted.	Page 5: Line 145 – 153
Search strategy	7	Present the full search strategies for all databases, registers and websites, including any filters and limits used.	Page 5: Line 154 – 183
Selection process	8	Specify the methods used to decide whether a study met the inclusion criteria of the review, including how many reviewers screened each record and each report retrieved, whether they worked independently, and if applicable, details of automation tools used in the process.	Page 5 and 6: Line 184 – 202
Data collection process	9	Specify the methods used to collect data from reports, including how many reviewers collected data from each report, whether they worked independently, any processes for obtaining or confirming data from study investigators, and if applicable, details of automation tools used in the process.	Page 6 and 7: Line 203 – 215
Data items	10a	List and define all outcomes for which data were sought. Specify whether all results that were compatible with each outcome domain in each study were sought (e.g. for all measures, time points, analyses), and if not, the methods used to decide which results to collect.	Page 7: Line 216 – 229
	10b	List and define all other variables for which data were sought (e.g. participant and intervention characteristics, funding sources). Describe any assumptions made about any missing or unclear information.	Page 7: Line 216 – 229
Study risk of bias assessment	11	Specify the methods used to assess risk of bias in the included studies, including details of the tool(s) used, how many reviewers assessed each study and whether they worked independently, and if applicable, details of automation tools used in the process.	Page 7: Line 230 – 234
Effect measures	12	Specify for each outcome the effect measure(s) (e.g. risk ratio, mean difference) used in the synthesis or presentation of results.	Page 7: Line 230 – 234
Synthesis methods	13a	Describe the processes used to decide which studies were eligible for each synthesis (e.g. tabulating the study intervention characteristics and comparing against the planned groups for each synthesis (item #5)).	Page 7: Line 230 – 234
	13b	Describe any methods required to prepare the data for presentation or synthesis, such as handling of missing summary statistics, or data conversions.	Page 7: Line 234 – 237
	13c	Describe any methods used to tabulate or visually display results of individual studies and syntheses.	Page 7: Line 234 – 237

PRISMA 2020 Checklist

Section and Topic	Item #	Checklist item	Location where item is reported
	13d	Describe any methods used to synthesize results and provide a rationale for the choice(s). If meta-analysis was performed, describe the model(s), method(s) to identify the presence and extent of statistical heterogeneity, and software package(s) used.	Page 7: Line 237 – 239
	13e	Describe any methods used to explore possible causes of heterogeneity among study results (e.g. subgroup analysis, meta-regression).	Page 7: Line 237 – 239
	13f	Describe any sensitivity analyses conducted to assess robustness of the synthesized results.	Page 7: Line 242 – 244
Reporting bias assessment	14	Describe any methods used to assess risk of bias due to missing results in a synthesis (arising from reporting biases).	Page 7: Line 230 – 234
Certainty assessment	15	Describe any methods used to assess certainty (or confidence) in the body of evidence for an outcome.	Page 7: Line 230 – 234
**RESULTS**			
Study selection	16a	Describe the results of the search and selection process, from the number of records identified in the search to the number of studies included in the review, ideally using a flow diagram.	Page 5 and 6: Line 184 - 202
	16b	Cite studies that might appear to meet the inclusion criteria, but which were excluded, and explain why they were excluded.	Page 4: Line 137 – 144
Study characteristics	17	Cite each included study and present its characteristics.	Page 8, 220 and 26: Table 1, 2 and 3
Risk of bias in studies	18	Present assessments of risk of bias for each included study.	Page 8, 220 and 26: Table 1, 2 and 3
Results of individual studies	19	For all outcomes, present, for each study: (a) summary statistics for each group (where appropriate) and (b) an effect estimate and its precision (e.g. confidence/credible interval), ideally using structured tables or plots.	Page 8, 220 and 26: Table 1, 2 and 3
Results of syntheses	20a	For each synthesis, briefly summarise the characteristics and risk of bias among contributing studies.	Page 8, 220 and 26: Table 1, 2 and 3
	20b	Present results of all statistical syntheses conducted. If meta-analysis was done, present for each the summary estimate and its precision (e.g. confidence/credible interval) and measures of statistical heterogeneity. If comparing groups, describe the direction of the effect.	Page 11: Line 281 – 290
	20c	Present results of all investigations of possible causes of heterogeneity among study results.	Page 8, 220 and 26: Table 1, 2 and 3
	20d	Present results of all sensitivity analyses conducted to assess the robustness of the synthesized results.	Page 7 - 41: Line 245 – 1373
Reporting biases	21	Present assessments of risk of bias due to missing results (arising from reporting biases) for each synthesis assessed.	Page 8, 220 and 26: Table 1, 2 and 3
Certainty of evidence	22	Present assessments of certainty (or confidence) in the body of evidence for each outcome assessed.	Page 8, 220 and 26: Table 1, 2 and 3
**DISCUSSION**			
Discussion	23a	Provide a general interpretation of the results in the context of other evidence.	Page 40: Line 1331 – 1339
	23b	Discuss any limitations of the evidence included in the review.	Page 41: Line 1362 – 1373
	23c	Discuss any limitations of the review processes used.	Page 41: Line 1374 – 1381

PRISMA 2020 Checklist

Section and Topic	Item #	Checklist item	Location where item is reported
	23d	Discuss implications of the results for practice, policy, and future research.	Page 40: Line 1348 – 1371
**OTHER INFORMATION**			
Registration and protocol	24a	Provide registration information for the review, including register name and registration number, or state that the review was not registered.	It was not recorded.
	24b	Indicate where the review protocol can be accessed, or state that a protocol was not prepared.	Page 4: Line 115 – 128<br>The PRISMA 2020 framework was used to improve transparency in the identification, screening, and selection of the literature considered for the review<br>(https://estech.shinyapps.io/prisma_flowdiagram/).
	24c	Describe and explain any amendments to information provided at registration or in the protocol.	No amendments to the registered protocol were mad
Support	25	Describe sources of financial or non-financial support for the review, and the role of the funders or sponsors in the review.	Not applicable
Competing interests	26	Declare any competing interests of review authors.	“The authors declare no conflicts of interest”
Availability of data, code and other materials	27	Report which of the following are publicly available and where they can be found: template data collection forms; data extracted from included studies; data used for all analyses; analytic code; any other materials used in the review.	The literature search was conducted using Scopus and Web of Science—selected for their extensive coverage of peer-reviewed research in the fields of food science, bioprocessing, separation technologies, and biomass valorization—using the account crodriguezy23@ucvvirtual.edu.pe<br>The search equation in Scopus and Web of Science was established as follows: ("Capsicum" OR "Capsicum annuum" OR "Capsicum frutescens" OR "Capsicum chinense" OR "Capsicum baccatum" OR "Capsicum pubescens" OR pepper OR "chili pepper" OR "chilli pepper" OR "hot pepper" OR "pepper seed*" OR "chili seed*" OR "chilli seed*" OR "Capsicum seed*") AND (extraction OR recovery OR separation OR fractionation OR purification OR isolation OR "ultra-sound-assisted extraction" OR "ultrasound assisted extraction" OR "microwave-assisted extraction" OR "microwave assisted extraction" OR "supercritical fluid extraction" OR "supercritical CO2" OR "enzyme-assisted extraction" OR "enzyme assisted extraction" OR "pulsed electric field*" OR "ohmic heating" OR "pressurized liquid extraction" OR "accelerated solvent extraction" OR "deep eutectic solvent*" OR "green extraction" OR "emerging extraction technolog*" OR "membrane separation" OR "membrane filtration" OR adsorption OR chromatography) AND (oil OR oils OR "seed oil*" OR lipid* OR fat* OR "fatty acid*" OR triglyceride* OR triacylglycerol* OR oleoresin* OR "oil extraction" OR "oil recovery" OR valorization OR valorisation OR biorefinery OR "cascade valorization" OR "cascade valorisation" OR "waste valorization" OR "waste valorisation" OR "by-product

PRISMA 2020 Checklist

Section and Topic	Item #	Checklist item	Location where item is reported
			valorization" OR "by-product valorisation" OR "circular bioeconomy" OR "circular economy" OR "value-added product*" OR "value added product*" OR "high-value compound*" OR "bioactive compound*" OR "functional ingredient*" OR nutraceutical* OR "food ingredient*").

From: Page MJ, McKenzie JE, Bossuyt PM, Boutron I, Hoffmann TC, Mulrow CD, et al. The PRISMA 2020 statement: an updated guideline for reporting systematic reviews. BMJ 2021;372:n71. doi: 10.1136/bmj.n71. This work is licensed under CC BY 4.0. To view a copy of this license, visit https://creativecommons.org/licenses/by/4.0/
